# Nanoscale electrochemistry in a copper/aqueous/oil three-phase system: surface structure–activity-corrosion potential relationships†

**DOI:** 10.1039/d0sc06516a

**Published:** 2020-12-22

**Authors:** Enrico Daviddi, Viacheslav Shkirskiy, Paul M. Kirkman, Mathew P. Robin, Cameron L. Bentley, Patrick R. Unwin

**Affiliations:** Department of Chemistry, University of Warwick Coventry CV4 7AL UK p.r.unwin@warwick.ac.uk; Lubrizol LTD Nether Ln Hazelwood DE56 4AN UK; School of Chemistry, Monash University Clayton Victoria 3800 Australia cameron.bentley@monash.edu

## Abstract

Practically important metal electrodes are usually polycrystalline, comprising surface grains of many different crystallographic orientations, as well as grain boundaries. In this study, scanning electrochemical cell microscopy (SECCM) is applied in tandem with co-located electron backscattered diffraction (EBSD) to give a holistic view of the relationship between the surface structure and the electrochemical activity and corrosion susceptibility of polycrystalline Cu. An unusual aqueous nanodroplet/oil (dodecane)/metal three-phase configuration is employed, which opens up new prospects for fundamental studies of multiphase electrochemical systems, and mimics the environment of corrosion in certain industrial and automotive applications. In this configuration, the nanodroplet formed at the end of the SECCM probe (nanopipette) is surrounded by dodecane, which acts as a reservoir for oil-soluble species (*e.g.*, O_2_) and can give rise to enhanced flux(es) across the immiscible liquid–liquid interface, as shown by finite element method (FEM) simulations. This unique three-phase configuration is used to fingerprint nanoscale corrosion in a nanodroplet cell, and to analyse the interrelationship between the Cu oxidation, Cu^2+^ deposition and oxygen reduction reaction (ORR) processes, together with nanoscale open circuit (corrosion) potential, in a grain-by-grain manner. Complex patterns of surface reactivity highlight the important role of grains of high-index orientation and microscopic surface defects (*e.g.*, microscratches) in modulating the corrosion-properties of polycrystalline Cu. This work provides a roadmap for in-depth surface structure–function studies in (electro)materials science and highlights how small variations in surface structure (*e.g.*, crystallographic orientation) can give rise to large differences in nanoscale reactivity.

## Abstract 2

Probing Cu corrosion in an aqueous nanodroplet/oil/metal three-phase environment revealed unique patterns of surface reactivity. The electrochemistry of high-index facets cannot be predicted simply from the low-index {001}, {011} and {111} responses.

## Graphical abstract

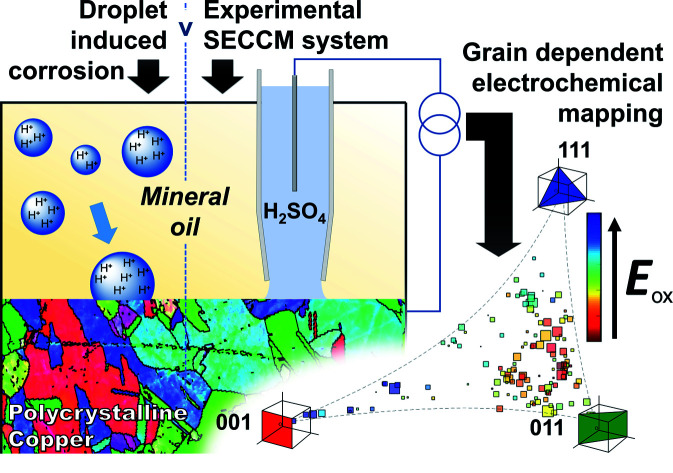

Corrosion has long been studied, as a significant concern and a costly issue (*ca.* 3% of the GDP of industrialised countries) for the modern world.^1,2^ For metals, in particular, electrochemical techniques, allied to complementary analytical and microscopy methods, play a central role in unveiling corrosion and corrosion protection mechanisms.^3–7^ However, a limitation of many experimental approaches is that the electrochemical perturbation (and measurement) is applied globally at a macroscopic electrode immersed in a bulk solution,^8^ but most corrosion processes are initiated and perpetuated at (sub)microscopic surface sites (*e.g.*, grain boundaries, inclusions, microscratches *etc.*).^9–14^ Mismatch between the scale of key corrosion phenomena and conventional electrochemical methods makes it difficult to unambiguously identify the key anodic/cathodic sites driving corrosion. This issue is compounded for the case of atmospheric corrosion,^15^ or corrosion in certain automotive/industrial environments (*vide infra*),^16,17^ which take place due to the action of small droplets on the surface in a confined system. Corrosion science needs electrochemical techniques that operate at the (sub)microscale, and allow activity and surface structure to be correlated commensurately at this scale.

Among the limited library of electrochemical techniques that can routinely operate at the (sub)microscale,^18,19^ scanning electrochemical cell microscopy (SECCM) is attracting significant attention.^20–22^ SECCM maps electrochemistry locally and directly *via* a nanoscale electrochemical meniscus cell (formed at the end of a fluidic probe) that makes measurements over an array of points (typically thousands of discrete areas) on an electrode (or other) surface. For polycrystalline surfaces, SECCM measurements are powerfully combined with co-located electron backscattered diffraction (EBSD), to elucidate nanoscale structure–activity, as exemplified by studies of various electrochemical processes at a range of polycrystalline materials, including Pt,^23–26^ Au,^27^ Pd,^28^ low carbon steel,^29–31^ Zn^32^ and boron-doped diamond.^33^

In addition to its high spatiotemporal resolution, the meniscus cell configuration of SECCM facilitates rapid reactant/product exchange with the surrounding environment, mimicking a gas diffusion electrode, with an enhanced flux of gases into the meniscus cell (*i.e.*, at the three-phase boundary).^24,27,34^ When operated in air, SECCM emulates the configuration of atmospheric corrosion, with gas exchange (*e.g.*, oxygen, O_2_) taking place across the liquid/gas interface of the meniscus in contact with a surface of interest. As recently reported, and expanded upon herein, SECCM can also be operated under oil immersion,^32,35^ which not only aids in confinement of the meniscus cell during prolonged measurements,^35^ but also opens up the possibility of studying the effect of oil-soluble species (*e.g.*, corrosion inhibitors, organic contaminants, redox mediators *etc.*) on local reactions at the solid/liquid/liquid interface with high spatial-resolution. This configuration is regaining interest for fundamental studies,^36,37^ as well as being of great practical importance (*e.g.*, phase-transfer reactions in industrial chemical processes, biology *etc.*).^38^

A key attribute of SECCM is that a number of conventional dynamic electrochemistry techniques (*e.g.*, potentiometry, amperometry and voltammetry) can be translated readily to the confines of the meniscus cell.^20,22,39^ Herein, the versatility of chronopotentiometry for local corrosion and electrochemical measurements is demonstrated. First, it is possible to make meniscus contact at zero applied current, corresponding to open circuit potential (OCP), which is measured. This corresponds to the corrosion (mixed) potential, where the rate of anodic dissolution of the metal (forming metal ions) and the rate of reduction of oxygen are balanced. Surface ion release under this condition is then analysed by subsequent “electrochemical titration” of a portion of the released metal ions, by applying a cathodic current and recording the resulting chronopotentiometric curve.^40^ This allows the evaluation of intrinsic corrosion susceptibility, *in situ*, with high spatial-resolution, for the entire range of crystallographic orientations of a polycrystalline metal (*i.e.*, revealed through co-located EBSD analysis). Chronopotentiometry measurements with and without O_2_ present, and the use of an anodic pulse to induce the anodic dissolution (as well as the cathodic measurements mentioned) allow all of the key electrochemical processes underpinning localised corrosion to be studied. The patterns of surface reactivity establish the intimate link between corrosion susceptibility, electrochemical kinetics and surface structure at the nanoscale.

## Experimental

### Chemical reagents and electrode materials

Sulphuric acid (H_2_SO_4_, Sigma Aldrich, Germany, 96%), copper(ii) sulphate (CuSO_4_, Merck, Germany, 99%), *n*-dodecane (C_12_H_26_, Sigma Aldrich, Germany, 99%) and potassium chloride (KCl, Honeywell, Germany, 99.5%) were used as supplied by the manufacturer without further purification. All solutions were prepared with ultrapure deionised water (resistivity = 18.2 MΩ cm at 25 °C).

The copper substrate was prepared from a 3 mm thick Cu foil (Goodfellow, U.K., 99.95%) that was cut into a square of ≈1.5 cm edge and hot mounted in a conductive carbon support (KonductoMet™, Buehler, U.S.A.) with a SimpliMet™ 3000 Mounting Press (Buehler, U.S.A.). The sample was mechanically polished on polishing cloths to a mirror finish, down to ≈50 nm roughness, using a series of aqueous diamond suspensions (9 μm MetaDi™ Supreme Diamond suspension on a Texmet C™ polishing pad and 3 μm MetaDi™ Supreme Diamond suspension on a Verdutext™ polishing pad, Buehler, U.S.A.), followed by colloidal silica for the last polishing step (0.02–0.06 μm MasterMet™ colloidal silica suspension on a ChemoMet™ polishing pad, Buehler, U.S.A.). The sample was then washed in deionised water and isopropanol and gently blown dry. Following preparation, the Cu substrate was aged under ambient conditions for 1 week before performing grain-resolved electrochemical analysis, in order to allow the natural layer of oxide to form (*vide infra*).^41^

The Ag/AgCl quasi-reference counter electrode (QRCE) was prepared by anodising an annealed silver wire (0.25 mm diameter, Goodfellow, U.K., 99.99%) in a saturated KCl solution. The potential of the QRCE was calibrated potentiometrically by immersing in the solution of interest (10 mM H_2_SO_4_) and monitoring the OCP *versus* a commercial 3.4 M Ag/AgCl electrode (ET072, eDAQ, Australia), which has a theoretical standard potential of +205 mV *vs.* the standard hydrogen electrode (SHE).^42^ As previously reported, the QRCE possessed a stable potential of *ca.* +180 mV *vs.* Ag/AgCl on the experimental (*i.e.*, hours) timescale.^43^

### Macroscale electrochemistry

Macroscopic electrochemical experiments were performed in a conventional three-electrode format on a commercial potentiostat/galvanostat (Femtostat, Gamry Instruments, U.S.A.). A commercial Cu macrodisk electrode (3 mm diameter, MF-2110, BASi, U.S.A.), commercial 3.4 M Ag/AgCl reference electrode (*vide supra*) and coiled platinum wire (0.5 mm thickness, ≈10 cm length) were used as working, reference and counter electrodes, respectively. When needed, the solution of interest was deaerated with nitrogen gas (N_2_) for at least 15 minutes, prior to experiments.

### Scanning electrochemical cell microscopy (SECCM)

**Fig. 1:**
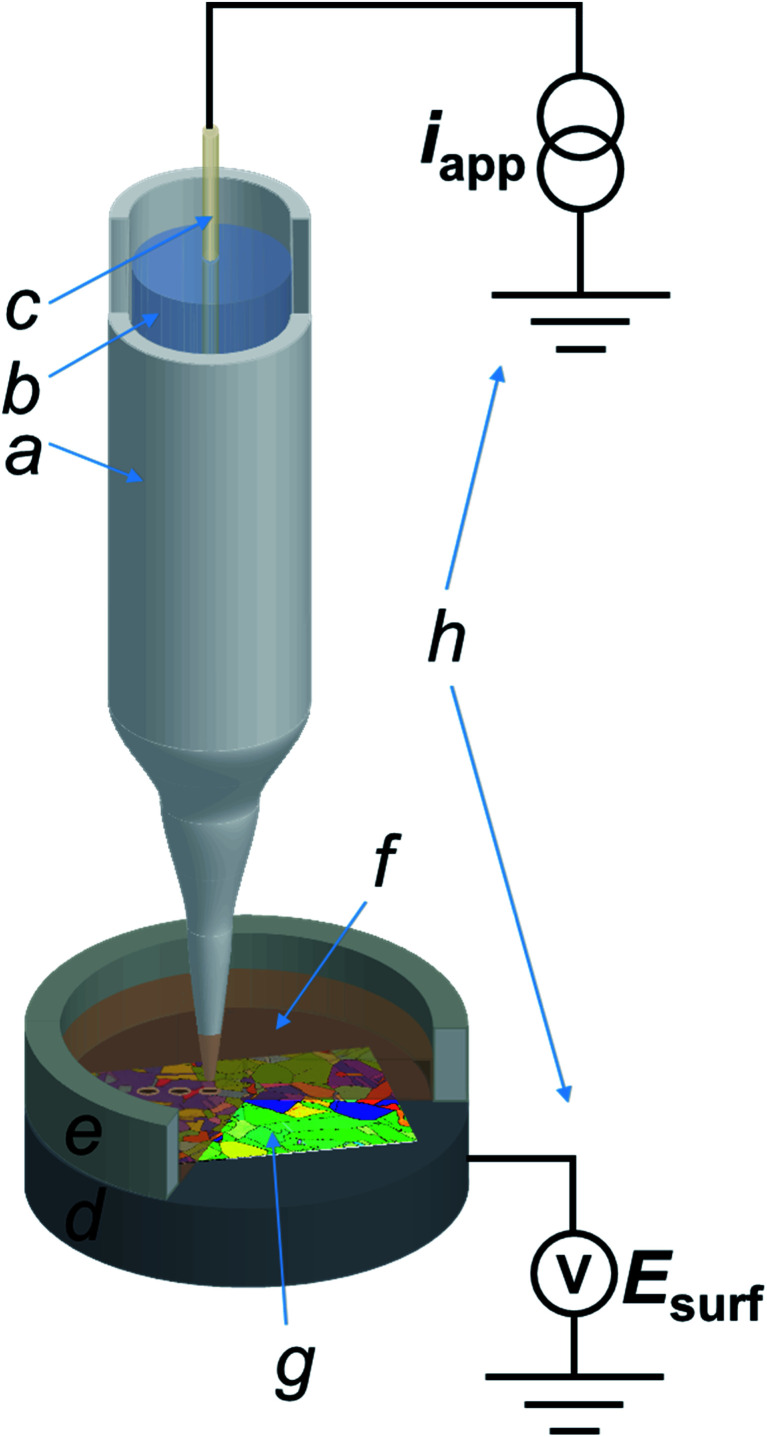
Scheme (not to scale) of the SECCM three-phase (Cu/aqueous/dodecane) setup employed in this work. The main components of the system are: (a) nanopipette probe; (b) electrolyte solution (H_2_SO_4_ 10 mM); (c) quasi-reference counter electrode (QRCE), connected to the galvanostat; (d) conductive carbon mounting for the sample, connected to the voltage measuring device; (e) epoxy resin well to contain dodecane; (f) dodecane layer covering (g) the polycrystalline copper (working electrode) surface and; (h) homebuilt galvanostat and potential measuring device.

All experiments in the SECCM configuration were performed on a home-built scanning electrochemical probe microscopy (SEPM) workstation, as detailed in previous works.^40,44^ The main components were an *x*–*y*–*z* piezoelectric positioner (P-611.3S, Physik Instrumente, Germany), a galvanostat (home-built, as described in a previous work,^40^ serving as voltage follower), a waveform generator (digital herein, utilized for applying the desired current to the galvanostat) and a data collection-instrument control system (FPGA card herein). The main setup is schematised in Fig. 1.

The nanopipette probe was made by pulling a filamented borosilicate glass capillary (GC120F-10, Harvard Apparatus, U.K.) to a sharp tip with a commercial CO_2_ laser puller (P-2000, Sutter Instruments, U.S.A.), with pulling parameters reported in the ESI, Section S.1.† The pulled nanopipette was filled with the electrolyte (10 mM H_2_SO_4_) and a silicone oil layer was added on the top of the solution in order to minimise evaporation from the back, as previously reported.^45^ The QRCE (described above) was then inserted into the nanopipette from the back and positioned approximately 3 cm from the tip end.^43^ Following preparation, the SECCM nanoprobe was mounted onto the piezoelectric positioner and moved into the initial position, *ca.* 50 μm above the Cu substrate (*vide supra*) by using coarse micropositioners (*x*–*y*–*z* movement), in tandem with an optical camera (PLB776U camera equipped with a 4× lens, Pixelink, Canada). After positioning, a layer of dodecane was placed onto the Cu substrate to a thickness that also covered the tip of the SECCM probe, and confined using a chemically-resistant epoxy resin well, as schematised in Fig. 1. Unless otherwise stated, the dodecane layer was exposed to the ambient air, meaning that oxygen (O_2_) was present in the SECCM droplet cell. When an O_2_-free environment was needed, an environmental cell was used, through which argon was flowed for at least an hour before and during experiments, as outlined previously.^32,46^

The entire SECCM setup was placed on a passive mechanical vibration isolator platform (Minus K Technology, U.S.A.), which was located in an aluminium Faraday cage equipped with heat sinks and acoustic foam to minimise mechanical vibration, electrical noise and thermal drift.

The SECCM experiments were performed in the chronopotentiometric mode, with a “hopping mode” imaging protocol, employing a home-built sensitive galvanostat with an ultralow input bias current, as previously described.^40^ The procedure consisted of approaching the nanopipette probe to the surface in a series of predefined points of a rectangular grid. The approach current was set to be zero (within the sensitivity range of the galvanostat, which had an instrumental error of ± 6 fA) so that, upon meniscus contact (only the droplet meniscus and not the nanopipette itself, made physical contact with the surface), the recorded potential was effectively the mixed corrosion potential, measured in conditions of net zero-current. This potential approximates closely to the open circuit potential (OCP) and it will be referred to as such, for brevity, noting its equivalence to the mixed corrosion potential. After the OCP measurement step, a chronopotentiometric pulse measurement was then performed to a desired value of applied current and the potential–time profile recorded for a defined period. The current (density) magnitude applied was sufficiently large (*i.e.* above the noise level of the electrochemical instrumentation) that the anodic and cathodic processes could be determined independently and in isolation, distinct from the measurement of the OCP. Subsequently, the nanopipette probe was retracted from the surface and moved laterally to begin the approach to the next point. The nanopipette translation speeds were 5 μm s^−1^ on approach, 20 μm s^−1^ on retract (by 1.5 μm) and 30 μm s^−1^ during lateral movement. The distance between each pixel (*i.e.*, the hopping distance) was 1 μm, larger than the diameter of the area wetted by the meniscus cell (*vide infra*), so as to ensure that each point measurement was independent of the last. At the beginning of each approach, the potential was set to the “overload” value of the FPGA card (+10 V), as previously described.^40^ A detectable decrease of more than 5 V from this value was set as the threshold to indicate when the meniscus had made contact with the surface. This signified that the electrochemical cell circuit was closed, and the abrupt change of signal allowed the tip movement to be immediately stopped and the chronopotentiometric pulse(s) to be applied and the response recorded.

The chronopotentiometric pulse data collected at each position were organised in order to create time-dependent two-dimensional maps (Movies S1 to S3†) of the recorded local working electrode potential as a function of time (*i.e.*, an electrochemical movie of *E*_surf_ in the imaged region as a function of time, where *E*_surf_ is the surface potential measured during SECCM). The current (*i*_app_) was applied at the QRCE and *E*_surf_ was measured at the substrate (working electrode), every 10 μs, with every digitally acquired point consisting in an average of 256 consecutive measurements, to give a digital data acquisition rate of 10 × (256 + 1) = 2.570 ms per point (note that one extra iteration was used to transfer the data to the host computer). Each electrochemical movie was collected on different spots of the same Cu sample. Two different nanopipette probes were employed for the anodic scan (Movie S1†) and the cathodic scans (Movies S2 and S3,† collected subsequently).

SECCM data were processed using custom scripts run on the Matlab R2018b (Mathworks, U.S.A.) software suite. The transition time (*τ*) was calculated at each pixel of Movies S2 and S3 by finding the time of the inflection point of the chronopotentiometric curve during the cathodic pulse. Data were plotted with OriginPro 2019 64 bit (9.60, OriginLab, U.S.A.) and Matlab R2018b (for the movies and all the 2D electrochemical maps) software packages. Note that all electrochemical images were plotted without any data interpolation.

### Surface characterisation

SEM and EBSD were performed with a Zeiss SUPRA FE-SEM (Zeiss, Germany), equipped with a Nordlys EBSD detector (Oxford Instruments, U.K.). SEM images were taken at an acceleration voltage of 5 keV with an InLens detector. The contact area of the meniscus cell with the surface of interest (polycrystalline Cu) was estimated by SEM imaging of footprints left on the surface after measurements, with measured diameters (precision better than ± 50 nm) being ≈600 nm (wetted area ≈ 1.6 × 10^−9^ cm^2^) for the anodic pulse experiments herein and ≈900 nm (wetted area ≈ 6.4 × 10^−9^ cm^2^) for the cathodic pulse experiments (ESI, Section S.2†). These aerial values were used to calculate applied current densities. Importantly, for any particular experimental run there was no noticeable influence of surface microstructure on the degree of wetting (ESI, section S.2†). Furthermore, the consistency of SECCM potential data within areas of the same surface structure, and for different times within an imaging run, indicates the consistency of the meniscus contact. EBSD mapping was carried out with an acceleration voltage of 20 keV, with the sample tilted 70° to the detector. EBSD data were processed using the HKL CHANNEL5 software (Tango, Oxford Instruments, U.K.) to extract inverse pole figure (IPFz) images and disorientation colouring maps (*i.e.*, disorientation angle between each couple of neighbouring pixels in the EBSD map) for each SECCM scan area.

## Results and discussion

### Copper electrochemistry in H_2_SO_4_ (pH 2): macroscale *vs.* microscale

Consulting the phase stability (potential/pH) diagram for copper,^47^ only one anodic reaction is possible at pH 2: the oxidation of Cu to form the soluble cupric ion (Cu^2+^):

1 Cu_(s)_ → Cu^2+^ + 2e^−^, *E*^0^ = 0.134 V *vs.* Ag/AgCl (3.4 M KCl)

where *E*^0^ is the standard electrode potential. eqn (1) is referred to as the Cu/Cu^2+^ process, herein. Note that although eqn (1) shows an overall 2 electron process, this is an oversimplification of the Cu oxidation process, which under most conditions occurs through the intermediate, Cu(i)_ads_, formed through coupled interfacial (electro)chemical reactions with complexing agents (*e.g.* Cl^−^ or SO_4_^2−^) or O_2_, in solution.^48–51^ An example of such reaction is the oxidation of the Cu(i) intermediate by dissolved oxygen:

2 Cu(i)_ads_ + ½ O_2_ → Cu^2+^ + ½H_2_O_2_,

which is thought to play a fundamental role in Cu dissolution in aerated acidic conditions.^51,52^ Indeed, stable Cu(i)_ads_ intermediates can be observed at pH > 5,^47^ in copper oxides, hydroxides or oxyhydroxides (collectively referred to as CuO_*x*_, herein), but these species are not thermodynamically stable at pH 2, given the following reaction:

3 CuO_*x*_ + (2*x*)H^+^ → Cu^2+^ + *x*H_2_O

although they may well play a role in multi-step Cu electrodissolution.

A number of cathodic processes need to be considered for the defined conditions, the most obvious being the oxygen reduction reaction [ORR, eqn (4)] and the hydrogen evolution reaction [HER, eqn (5)]:

4 O_2_ + 2H^+^ + 2e^−^ → H_2_O_2_, *E*^0^ = 0.322 V *vs.* Ag/AgCl (3.4 M KCl) at pH = 2

5 2H^+^ + 2e^−^ → H_2_, *E*^0^ = −0.323 V *vs.* Ag/AgCl (3.4 M KCl) at pH = 2

Note that in acidic media, the ORR reportedly follows the 2e^−^ pathway to form H_2_O_2_ as the main product.^51,53^ From the *E*^0^ values of eqn (1) and (4), Cu dissolution occurs spontaneously upon immersion in aerated solution (pH = 2), as per the following equation:

6 Cu + O_2_ + 2H^+^ → Cu^2+^ + H_2_O_2_

Eqn (6) is the Cu corrosion reaction that occurs at the OCP. Thus, for the conditions of the present measurements, a third cathodic process that needs to be considered is the reduction (electrodeposition) of Cu^2+^ [liberated during eqn (6)] to Cu_(s)_ [*i.e.*, the reverse of eqn (1)], referred to as the Cu^2+^/Cu process, herein. The important role of HSO_4_^−^/SO_4_^2−^ through surface complexation, as well as the intercalation of these ions within Cu adlayers during the deposition process, has previously been reported.^54–57^

Constant current chronopotentiometry is a powerful approach for separating electrochemical processes that occur at similar potentials [*e.g.*, eqn (1), (4) and (5)],^58^ and this technique has recently been implemented in SECCM.^40^ When a constant current condition is imposed, the working electrode (Cu herein) adopts the potential necessary to drive the most favourable redox (electron-transfer) reaction at the rate necessary to support the applied current. If conditions change so that the applied current can no longer be supported by such redox reaction (*e.g.*, due to depletion of redox species at the electrode surface), the working electrode potential shifts, at the transition time (*τ*), to the value necessary to carry out the next most favourable reaction at the imposed rate. By plotting the measured potential as a function of time, closely spaced redox processes can be resolved as distinct potential plateaus.^59^

**Fig. 2:**
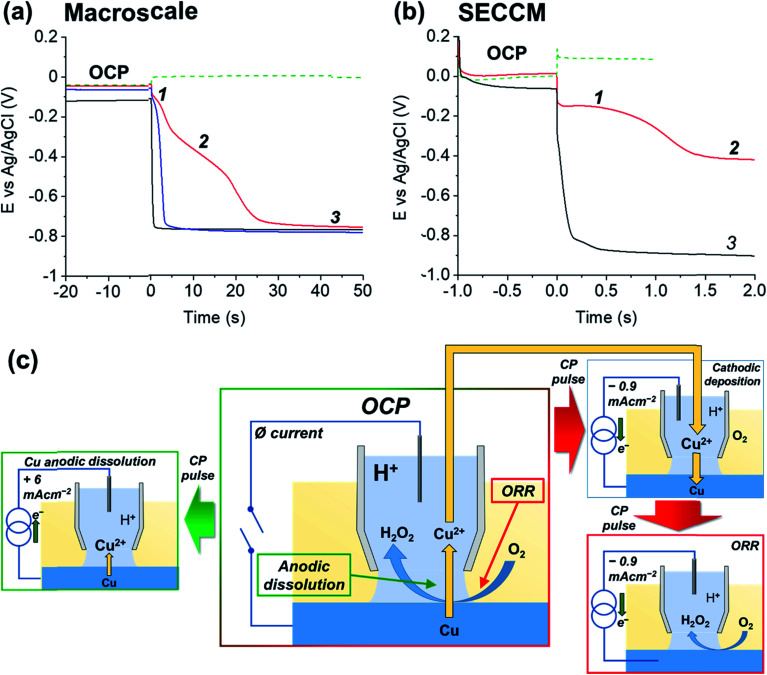
Anodic and cathodic chronopotentiometric (*E*–*t*) curves obtained from polycrystalline Cu (10 mM H_2_SO_4_, pH = 2) at (a) a Cu macrodisk electrode (area, *A* = 0.071 cm^2^) and (b) in the three-phase SECCM configuration. In (a), traces are shown for anodic (green) and cathodic (red, blue and black traces) *I*_app_ pulses of 0.1 mA cm^−2^ magnitude preceded by 20 s OCP pre-equilibrium. The experiments were carried out in aerated H_2_SO_4_ (red and green curves), deaerated H_2_SO_4_ (black curve) and deaerated H_2_SO_4_ containing 0.2 mM CuSO_4_ (blue curve). In (b), anodic (green trace) and cathodic (red trace) *I*_app_ pulses of 6 and 0.88 mA cm^−2^, respectively at a polycrystalline Cu foil electrode (immersed in dodecane) were preceded by 1 s OCP pre-equilibrium. The contact area (“footprint”) of the meniscus cell was 1.6 × 10^−9^ and 6.4 × 10^−9^ cm^2^ for the anodic and cathodic *I*_app_ pulse, respectively. The anodic and cathodic *E*_surf_–*t* curves were obtained by averaging 10 287 and 11 556 individual measurements, respectively. The numbers on the curves in (a) and (b) define the specific cathodic processes described in the text. (c) Schematics of the reactions portrayed in both the anodic (represented by the green arrows) and cathodic (represented by the red arrows) potentiometric analyses in the SECCM configuration. The Cu^2+^ released during the OCP step (mixed potential due to the Cu/Cu^2+^ reaction and ORR) is redeposited during the first part of the cathodic pulse (with the connection between the two processes represented by the yellow arrow).

To demonstrate the merit of the technique for resolving the Cu/Cu^2+^, ORR and HER processes [eqn (1), (4) and (5), respectively], a comparative chronopotentiometry study was carried out at the macroscale (*i.e.*, in bulk solution) and microscale (*i.e.*, in the SECCM configuration, Fig. 1a), as shown respectively in Fig. 2a and b. Note that all current pulses were preceded by an equilibrium pulse at the OCP (*i.e.*, *i*_app_ set to zero), which is represented on the resulting *E*–*t* plots at “negative times”. The anodic current pulse in bulk (Fig. 2a, green dashed line) and the SECCM configuration (Fig. 2b, green dashed line) results in a constant potential plateau corresponding to Cu oxidation *via* the reaction shown in eqn (1). For electrodissolution, the potential remains at a constant value (*i.e.*, no transition is observed) because of the constant supply of reactant (*i.e.*, Cu).

During the cathodic current pulse in bulk aerated solution (Fig. 2a, red line), three processes (plateaus) are observed at the following potentials: (1) ≈ −0.1 V, (2) ≈ −0.4 V and (3) ≈ −0.75 V *vs.* Ag/AgCl. Thus, the three cathodic processes discussed above are easily discriminated using chronopotentiometry. By performing control experiments in deaerated solution, where only the HER is possible (Fig. 2a, black line) and deaerated solution spiked with 0.2 mM CuSO_4_, where both Cu^2+^/Cu and the HER are possible (Fig. 2a, blue line), plateaus (1), (2) and (3) are assigned to Cu^2+^/Cu [reverse of eqn (1)], ORR [eqn (4)] and HER [eqn (5)], respectively. It should be re-iterated, that in bulk aerated solution (*i.e.*, Fig. 2a, red line), the Cu^2+^ that gives rise to plateau (1) is released into solution during the OCP pre-pulse, from the acid etching of Cu(s) in the presence O_2_ [*i.e.*, eqn (6)]. These results are consistent with previous work on chronopotentiometric Cu deposition in H_2_SO_4_ at the macroscale.^60^

The corresponding SECCM chronopotentiometric pulse experiment carried out on a polycrystalline Cu surface (immersed in dodecane) (Fig. 1) produced some different features to the macroscopic case. Each individual *E*_surf_–*t* experiment was limited to 3 seconds (1 second OCP, 2 seconds current pulse) to maintain meniscus cell stability (*i.e.*, minimise surface wetting) and ensure reasonable imaging times when creating surface arrays of data.^40^ Taking into consideration that *τ* ∝ *I*_app_^−2^ (*vide infra*), a larger applied current density (*I*_app_) value of 0.88 mA cm^−2^ (*i*_app_ = 5.65 pA, see ESI Section S.2† for the measured wetting area) had to be employed in the SECCM configuration (*c.f.* 0.1 mA cm^−2^ in the macroscale experiment) to observe both the Cu^2+^/Cu and ORR processes on this timescale. A typical *E*_surf_–*t* curve obtained by averaging 11 556 individual measurements, each at a separate location across a polycrystalline Cu surface, is shown in Fig. 2b (red line). Analogous to the case in bulk solution (*vide supra*), plateau (1), the Cu^2+^/Cu process, occurs at *ca.* −0.15 V, which undergoes a transition (at time *τ* ≈ 1.1 s) to plateau (2), the ORR, at *ca.* −0.42 V.

In order to verify the nature of such plateaus, a comparison with an SECCM analysis in de-aerated atmosphere (Fig. 2b, black curve, average of 625 individual measurements across a Cu surface), obtained by applying *I*_app_ = 0.95 mA cm^−2^ (*i*_app_ = 30 pA, note the larger wetting radius of the SECCM meniscus cell under these conditions, ESI, Section S.2†). As in the macroscopic case, in the absence of O_2_, *E*_surf_ shifts rapidly and settles directly on a plateau compatible with process (3) (≈−0.85 V), *i.e.*, the HER. Note that in the aerated SECCM configuration, the final transition to the HER was not observed, even when a longer pulse time was applied (*i.e.*, tens of seconds timescale). As explored in detail in the ESI, Section S.3,† this is due to the high flux of O_2_ into the SECCM droplet from the oil phase. In fact, the oil phase acts as an infinite reservoir of O_2_, compressing the diffusion layer and effectively reaching a steady state where the condition for the transition (O_2_ concentration at the electrode being zero) is never met;^40,61,62^ this happens because *I*_app_ (0.88 mA cm^−2^) is *ca.* 100 times smaller than the steady-state current for the ORR (84 mA cm^−2^, as calculated in the ESI, Section S.3†).

Note that the absence of a plateau (1) in the de-aerated case (Fig. 2b, black curve) indicates that the amount of Cu^2+^ released by oxide dissolution, eqn (3), is negligible (undetectable) compared to that released from the corrosion of Cu, eqn (6), in aerated solution (Fig. 2b, red curve). Although this is inferred from the surface average behaviour (Fig. 2b, black curve), it should be noted that under de-aerated conditions, a rapid transition is consistently observed during all single point measurements, with no significant variation in the transition time (<0.1 s) between the individual grains examined. Hence it is assumed that the amount of Cu^2+^ released from oxide dissolution is negligible on all grains. Thus, as explored below, by ‘titrating’ the Cu^2+^ released during eqn (6), by application of a cathodic current, *τ* can effectively serve an *in situ* indicator of the extent of the Cu corrosion that occurs with zero applied current (*i.e.*, at OCP). To illustrate this, and further emphasise all of the processes relevant to Cu corrosion in acid media [*i.e.*, Cu/Cu^2+^, Cu^2+^/Cu and the ORR], a schematic is presented in Fig. 2c of the key processes that may occur in SECCM at OCP and during the cathodic and anodic pulses.

As alluded to above, *τ* is the time at which the concentration of the reactant (Cu^2+^ in this case) reaches zero at the electrode surface, and can be expressed by the Sand equation under conditions of planar (one-dimensional) mass-transport:^63^7
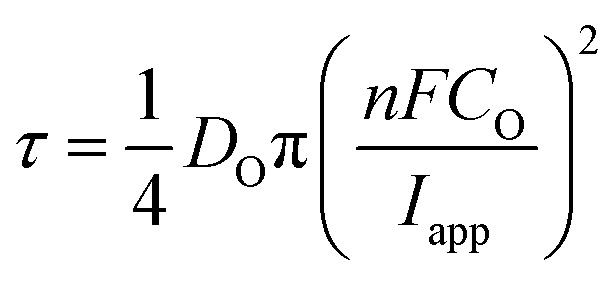
where *C*_O_ is the bulk concentration of the reactant, *D*_O_ is the diffusion coefficient and *n* is the number of electrons exchanged in the reaction. As showed previously,^40^ under the quasi-radial mass-transport conditions of SECCM, *τ* can be approximated by eqn (7) when the magnitude of *I*_app_ is several times larger than the magnitude of the steady-state limiting current density (which, as explored above, is not the case for the ORR reaction), analogous to the case at ultramicroelectrodes.^61,62^ In the present case, *D*_O_ and *n* have values of ≈7.2 × 10^−6^ cm^2^ s^−1^ (ref. 64) and 2, respectively (reasonably assumed independent of grain orientation). In addition, as shown in the ESI, Section S.2,† there is no significant variation in the meniscus cell wetting from point-to-point within the scan, and thus *I*_app_ possesses a constant value of ≈0.88 mA cm^−2^. As such, any variation in *τ* between grains reflects variation in the effective concentration of solubilised Cu^2+^, arising from the corrosion of Cu_(s)_ during the OCP pre-equilibrium step [*i.e.*, eqn (6)], effectively functioning as an indirect method of titration of the released ions. Herein, the relationship between *τ* and the relevant corrosion reactions (*vide supra*) is used, qualitatively, to fingerprint regions of the surface where corrosion is most facile (longer *τ*); eqn (7) does not apply quantitatively, as the Cu^2+^ concentration is transient and local.

In reference to eqn (1) and (4), the key factor for evaluating the ease of a reaction on a certain point of the surface is *E*_surf_, which is dependent on both thermodynamic and kinetic factors:^59^

8 *E*_surf_ = *E*^0^′ + *Q*

where *E*^0^′ (≈*E*^0^) is the formal potential of the main involved electrochemical step, and *Q* is dependent on *I*_app_ and the interplay of electrochemical kinetics (rate constant, concentration)-mass transport. As *I*_app_, and mass transport conditions are constant throughout an SECCM scan, variations in the measured *E*_surf_ informs on the local thermodynamics and kinetics of the processes, analogous to chronopotentiometry at ultramicroelectrodes.^61^

### Grain-dependent anodic processes: copper anodic oxidation reaction

**Fig. 3:**
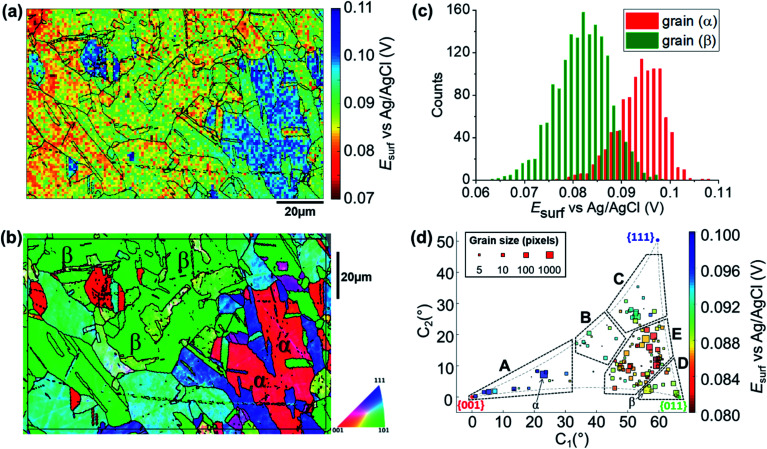
(a) *E*_surf_ map of the Cu/Cu^2+^ (Cu oxidation) process on a polycrystalline Cu foil electrode immersed in dodecane, obtained in the three-phase SECCM configuration with *I*_app_ = +6.25 mA cm^−2^. The nanopipette probe contained aerated 10 mM H_2_SO_4_ and contacted an area of the surface of 1.6 × 10^−9^ cm^2^. The map was extracted at time 0.2 s of the ESI, Movie S1.† (b) Crystallographic orientation map, IPFz (Inverse Pole Figure normal to the *z* axis) obtained with EBSD. Grains marked α and β are representative of {001}//ND and {011}//ND orientations, respectively. The grain boundaries in (b) are overlaid on the *E*_surf_ map in (a). (c) Statistical distribution of *E*_surf_ extracted from grains α and β, marked in (b). (d) Full grain orientation correlation analysis of *E*_surf_ at 0.2 s, extracted from (a) (and ESI, Movie S1†), *versus* the average grain orientation, extracted from (b), projected onto a 2D plot, as explored in the ESI, Section S.6.† The labels are used to group particular regions for the textual discussion. Details of the data extracted for each single grain can be also found in the ESI, Section S.7 (Fig. S9 and Table S2†).

To map the grain-dependence of the Cu/Cu^2+^ process (*i.e.*, copper electrodissolution) an anodic waveform comprising of OCP for 1 second, followed by *I*_app_ ≈ +6.25 mA cm^−2^ (with an applied current of 10 pA and an average wetting area of 1.6 × 10^−9^ cm,^2^ determined from the SEM image of the scanned area, ESI Fig. S1†) for 1.5 seconds was used at each position of the surface of polycrystalline Cu. A spatiotemporally-resolved electrochemical movie of Cu/Cu^2+^ activity (maps of potential as a function of time) is included in ESI, Movie S1 (details in ESI, S.4†). A single frame of Movie S1,† taken at a time of 0.2 s into the anodic pulse, is shown in Fig. 3a. Comparison of these data with the co-located crystallographic orientation (EBSD) map in Fig. 3b reveals the strong crystallographic dependence of the Cu/Cu^2+^ process. As part of the measurement protocol (*vide supra*) OCP data were collected at the same locations, and a strong grain dependency was also observed, as shown in the ESI (section S.5).† A discussion of the grain-dependent OCP characteristics is presented for data collected with the cathodic pulse SECCM scan (*vide infra*).

Due to the large number of independent *E*_surf_–*t* measurements made on individual grains (*e.g.*, see Fig. 3a), structure-dependent distributions of electrochemical activity can be extracted and statistically analysed. For example, a comparison between the distribution of *E*_surf_ extracted from the significant grains marked α and β in Fig. 3b, is shown in Fig. 3c. Grains α and β possess orientations within a few degrees of the {001}//ND and {011}//ND orientations, respectively, and possess average *E*_surf_ values (taken at the maximum of the normal distributions in Fig. 3c) of +0.095 V (std. dev. = 0.010 V) and +0.082 V (std. dev. = 0.012 V). Despite this relatively high variance, and the resulting partial overlapping of the two distributions, there is a clear difference in Cu oxidation susceptibility (of ≈13 mV) between the two grains, highlighting the strength of SECCM for resolving even small differences in nanoscale structure–activity.^18,22^

Comparing representative grains, such as α and β in Fig. 3b, provides insight into the structure-dependent electrochemical activity of a surface.^30,32^ It should be noted, however, that even these “representative grains” are not perfectly coincident with the ideal low-index grains, {001}//ND, {011}//ND and {111}//ND, which are often the subject of macroscopic single-crystal experiments and theoretical studies.^65–68^ To gain a more complete view of structure-dependent Cu oxidation susceptibility, a two-dimensional (2D) representation of the crystallographic-orientation was developed, analogous to the inverse pole figure in the *z* direction (IPFz) colour key (*e.g.*, see Fig. 3b). Details of the development and implementation of this 2D projection are given in the ESI, Section S.6.† In essence, the crystallographic orientations within a scan area, determined by EBSD analysis, are plotted in Cartesian space (*x*–*y* coordinates denote the orientation of the grain relative to the ideal low-index orientations) and the electrochemical activity (*i.e.*, *E*_surf_), determined by SECCM analysis, is plotted on a separate colour scale. In this way, the crystallographic orientation and electrochemical activity, indicated by the *x*–*y* position and colour of each point, respectively, can be readily visualised and compared for many surface orientations of a polycrystalline surface. This kind of extended analysis has never been performed on such a wide range of crystallographic orientations; in fact, the analysis of polycrystalline surfaces has involved, so far, mainly the specific comparison of selected grains, usually representative of the low index grains of the crystallographic system of the material of interest.^9,10,14,24,28,29,31,32,69^ Therefore, this work represents a significant advance in the use of SECCM to study the relationship between electrode surface structure and activity.

A correlative plot of *E*_surf_*versus* grain orientation, constructed from the SECCM and EBSD datasets (Fig. 3a and b, respectively), is shown in Fig. 3d. In order to compare to the corrosion-related processes, five distinct areas within which the activities are broadly similar are demarcated A to E in Fig. 3d: (A) Grains close to the {001}//ND plane, 0.096 < *E*_surf_ < 0.1 V; (B) the band of grains around the {225}//ND grain, 0.09 < *E*_surf_ < 0.094 V; (C) grains toward the {111}//ND orientation, 0.09 < *E*_surf_ < 0.094 V; (D) grains close to the {011}//ND orientation, 0.088 < *E*_surf_ < 0.09 V and; (E) the band of grains located between B, C and D, *E*_surf_ < 0.088 V. Notably, area E, of intermediate orientation, is the most susceptible to oxidation and is in an area of the plot that is closest to the {011}//ND plane. Note that the grains that lay at the boundaries of the distinct areas of activity, such as grain β (Fig. 3b), which is between areas D and E, tend to show intermediate electrochemical behaviours, making the transition between neighbouring areas of the plot relatively smooth.

### Grain-dependent cathodic processes: oxygen reduction reaction

**Fig. 4:**
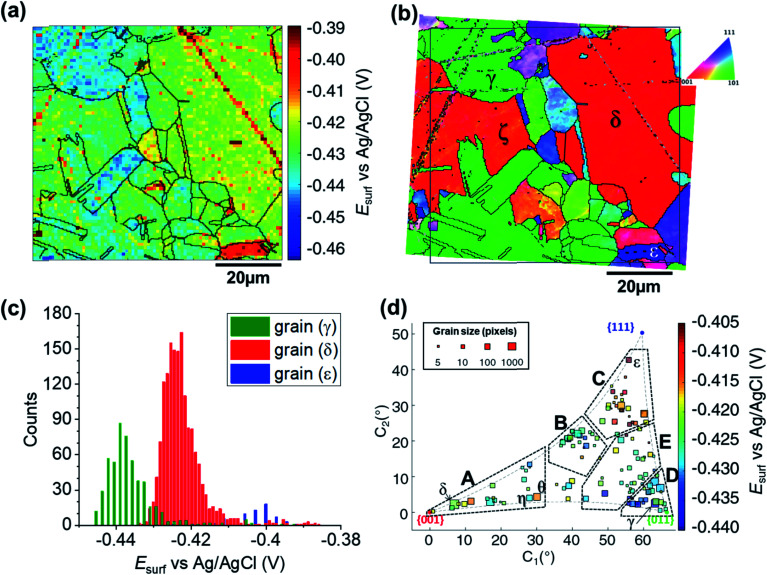
(a) Surface potential (*E*_surf_) map of the ORR process on a polycrystalline Cu foil electrode immersed in dodecane, obtained in the SECCM configuration with *I*_app_ = −0.88 mA cm^−2^. During these measurements, the nanopipet probe contained aerated 10 mM H_2_SO_4_ and contacted an area of 6.4 × 10^−9^ cm^2^. The map was extracted at time 2 s of ESI, Movie S2.† (b) Crystallographic orientation map, IPFz obtained with EBSD. Grains marked γ, δ and ε are representative of {011}//ND, {001}//ND and {111}//ND orientations, respectively. The grain boundaries from (b) are overlaid on the *E*_surf_ map in (a). (c) Statistical distribution of *E*_surf_ in three representative grains, designated γ, δ and ε in (b). (d) Full grain orientation correlation analysis of *E*_surf_ at 2 s, extracted from (a) and Fig. S10a (ESI, Section S.7†), respectively for Movie S2 and S3,†*versus* the average grain orientation, extracted, respectively, from (b) and Fig. S10b (ESI, section S.7†), and projected onto a 2D plot. Details of the data extracted for each single grain can be found in the ESI, Section S.7 (Fig. S11 and Table S3†).

The SECCM chronopotentiometric cathodic waveform comprised of OCP for 1 second, followed by *I*_app_ = −0.88 mA cm^−2^ for 2 seconds (consisting in an applied current of 0.565 pA oven an average wetting area of 6.4 × 10^−9^ cm^2^, measured from the SEM image of the scanned area, ESI Fig^.^ S2 and S3†). Spatiotemporal-resolved electrochemical movies of cathodic activity are included in the ESI, Movie S2 and S3† (obtained for two different areas of the Cu working electrode surface). A single frame of Movie S2,† taken at a time when *E*_surf_ is dictated by the energetics (*i.e.*, thermodynamics/kinetics) of the ORR (*i.e.*, *t* = 2 s in Fig. 2b), is shown in Fig. 4a (note that the Cu^2+^/Cu process is explored separately, below). Comparison of Fig. 4a with the co-located EBSD map presented in Fig. 4b reveals a clear correlation between *E*_surf_ and crystallographic orientation. To illustrate this, three grains were selected from Fig. 4b, labelled γ, δ and ε. γ is representative of the {011}//ND family, with an average orientation of {0.03 0.67 0.73}//ND; δ is representative of the {001}//ND family, with an average orientation of {0.067 0.042 1.0}//ND; and ε is representative of the {111}//ND family, with an average orientation of {0.54 0.53 0.66}//ND. Comparing the statistical distribution of the *E*_surf_ associated with the ORR process measured at each position within these grains (Fig. 4c) gives the following values: *E*_surf,γ_ = −0.439 V (st. dev. 0.008 V), *E*_surf,δ_ = −0.424 V (st. dev. 0.007 V) and *E*_surf,ε_ = −0.401 V (st. dev. 0.007 V). The inter-grain variation in *E*_surf_ (*ca.* 0.05 V) is similar to that measured for the Cu/Cu^2+^ process (*ca.* 0.04 V, Fig. 3a) while the intra-grain variation is around three times smaller, making clearer the difference between these example grains.

Considering the *E*_surf_ – crystallographic orientation correlation plot (Fig. 4d), a highly complex grain dependence can be observed. For sake of comparison with the Cu/Cu^2+^ process analysed earlier, five activity areas (named A to E) are highlighted in Fig. 4d, in the same positions as the ones in Fig. 3d. Area C, comprising the grains that are closest to the {111}//ND orientation (*e.g.*, grain ε) possess the highest ORR activity (−0.420 < *E*_surf_ < −0.405 V), while area D, with the grains that are closest to the {011}//ND orientation (*e.g.*, grain γ) possess the lowest ORR activity (*E*_surf_ < −0.430, with a couple of exceptions). The other three areas have intermediate values of *E*_surf_, with areas B and E having −0.430 < *E*_surf_ < −0.420 V, and area A in the range – 0.430 V to −0.415 V. It is worth noting that the lowest-activity grains in area D are not exactly coincident with {011}//ND, but rather in a group lying at lower values of *C*_1_ (≈58° instead of ≈66° of the {011}).

For comparison with literature studies on single crystals, comparing grains γ, δ and ε, ORR kinetics increases in the order Cu{011} < Cu{001} < Cu{111}. The relative ORR rates on the Cu{111} and Cu{001} facets are in agreement with previous voltammetric studies on macroscopic single crystal electrodes,^66,70^ which ascribed variations in the ORR kinetics and/or reaction pathway with grain structure to the adsorption of spectator species, specifically (bi)sulphate (*i.e.*, HSO_4_^−^ and SO_4_^2−^) in aqueous H_2_SO_4_. In other words, the structure (and potential) dependent adsorption of (bi)sulphate anions blocks catalytic active sites for the adsorption of O_2_ molecules, inhibiting the ORR in a grain-dependent manner. While analogous experimental literature is not available on Cu{011}, it is worth noting that this crystal plane is considered to have strongest binding for dissociated oxygen.^71–73^ As Cu is on the descending branch of the classical volcano plot for the ORR (noting that the literature focuses more on the 4e^−^ reduction; studies of Cu amalgams suggest the same trend for the 2e^−^ process),^74,75^ it follows that an increased binding strength would result in more sluggish ORR kinetics, consistent with the present measurements, and highlighting the value of SECCM-EBSD pseudo-single crystal studies in being able to investigate a wide range of surface structures well beyond what is possible with classical single crystal studies.

According to previous STM and theoretical studies,^68,72,76–78^ the interaction of oxygen with Cu{001} and Cu{011} grains causes significant surface reconstruction, which is not observed on {111}//ND surfaces. This may also explain why grains that are closer to the {111}//ND orientation appear to possess the fastest ORR kinetics, by lowering the binding strength (*i.e.*, towards the peak of the volcano plot).

**Fig. 5:**
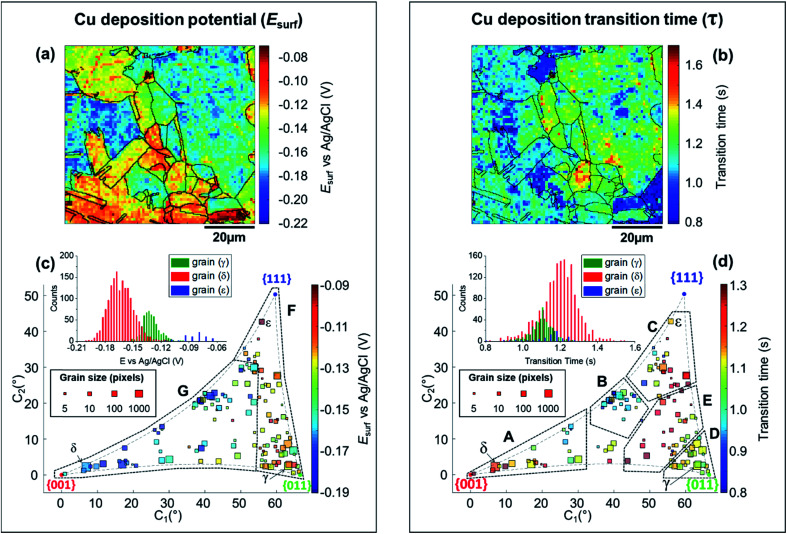
(a) *E*_surf_ map for the Cu^2+^/Cu process (Cu electrodeposition) on a polycrystalline Cu foil electrode immersed in dodecane, obtained with SECCM with *I*_app_ = −0.88 mA cm^−2^. The nanopipette probe contained aerated 10 mM H_2_SO_4_ and contacted an area of 6.4 × 10^−9^ cm^2^. The map was extracted at time 0.25 s of Movie S2.† (b) Map of *τ*_Cu^2+^/Cu_, extracted from Movie S2.† The grain areas determined from Fig. 4b are overlaid on the maps in (a) and (b). (c and d) Full grain orientation correlation analysis of (c) *E*_surf_ at 0.25 s and (d) *τ*_Cu^2+^/Cu_, extracted from (a) and Fig. S10c, and (b) and Fig. S10d,† respectively, with the average grain orientation extracted from Fig. 4b and S10b.† The insets in (c and d) show the distributions of *E*_surf_ and *τ*_Cu^2+^/Cu_, extracted from grains γ, δ and ε, marked in Fig. 4b. Details of the data extracted for each grain can be found in the ESI, Section S.7 (Fig. S11 and Table S3†).

It is also interesting to note that individual high-index grains shown in Fig. 5c can present reactivity that is distinct from neighbouring grains of similar structure. For instance, consider the two grains labelled η and θ in Fig. 4d, area A. While these grains differ by only 2.12° in the 2D projection (their average Miller indexes are {0.306 0.036 0.951} and {0.329 0.040 0.944}, respectively), grain θ possesses significantly higher activity (*E*_surf_ = −0.415 V) compared to η (*E*_surf_ = −0.427 V) and the surrounding grains (*E*_surf_ average of neighbouring grains with less than 10° difference in orientation being also −0.427 V). Such a difference, which is found in two grains that are relatively big and of comparable size (thus relatively immune to random statistical variations), demonstrates that the grain dependency of surface electrochemical reactions cannot be interpreted simply in terms of a surface structure that is a composite of individual low-index grains.

### Grain-dependent cathodic processes: copper deposition reaction

The analysis of the part of the cathodic chronopotentiometric pulse transient concerning the Cu^2+^/Cu process [plateau (1) in Fig. 2b] focuses on two fundamental parameters, *E*_surf_ and *τ*. These parameters carry different and complementary information, with *E*_surf_ indicating on the energetics (kinetics/thermodynamics) of the reaction, and *τ* indicating on the amount of Cu^2+^ released into solution during the OCP stage [*i.e.*, due to corrosion, as per eqn (6)]. Fig. 5a shows a single frame of Movie S2,† taken at a relatively early time, *t* = 0.25 s when *I*_app_ is solely supplied by the Cu^2+^/Cu process, while Fig. 5b shows a spatially-resolved map of *τ*_Cu^2+^/Cu_ (derived from the *E*_surf_ data in Movie S2†). Comparing Fig. 5a and b (and Movie S2†) with the co-located EBSD map in Fig. 4b, shows that there is a clear correlation between electrochemical activity (as reflected in both *E*_surf_ and *τ*) and surface crystallographic orientation. This is also clear from the 2D plots of *E*_surf_ and *τ versus* crystallographic orientation, shown in Fig. 5c and d, respectively.

Focusing on the grain-dependent *E*_surf_ characteristics (Fig. 5c), two distinct areas can be identified on the plot, marked F and G, listed below in order of decreasing ease of reduction. (F) Grains with orientations between the {011}//ND and {111}//ND low-index planes, −0.13 < *E*_surf_ < −0.09 V and; (G) grains close to the {001}//ND orientation (specifically the ones with *C*_1_ < 50° and *C*_2_ < 30°), −0.19 < *E*_surf_ < −0.15 V. Notably, grains close to the {001}//ND orientation result in both the highest anodic (Cu/Cu^2+^, Fig. 3d) and the lowest cathodic potentials (Cu^2+^/Cu, Fig. 5c), and thus the multistage Cu dissolution and Cu^2+^ deposition processes are not simply related. As introduced above, the intermediate Cu(i)_ads_ species on the surface play a fundamental role in the dissolution process, with its subsequent oxidation to Cu(ii) considered to be the rate determining step,^48,49^ and being highly affected by the presence of O_2_ in solution.^52,79,80^ Conversely, for the re-deposition process (*i.e.*, Cu^2+^/Cu), sulphate complexation and intercalation is poised to play a major role.^54–57^ The difference in mechanism may explain the different grain dependencies of the dissolution and deposition processes.

*E*_surf_ associated with the Cu^2+^/Cu process also shows significantly higher range of values between grains (≈0.15 V difference) compared to 0.04 V (for Cu/Cu^2+^) and 0.05 V (ORR); compare Fig. 5a, 3a and 4a, respectively. Comparing the statistical distribution of the *E*_surf_ associated with the Cu^2+^/Cu process measured at each position within the three grains highlighted in Fig. 4b, γ, δ and ε, (Fig. 5d, inset) gives the following values: *E*_surf,γ_ = −0.132 V (st. dev. 0.014 V), *E*_surf,δ_ = −0.164 V (st. dev. 0.026 V) and *E*_surf,ε_ = −0.082 V (st. dev. 0.020 V). It is worth noting that although there is significant inter-grain variations in *E*_surf_ (*i.e.*, compare γ, δ and ε in Fig. 5c, inset), the intra-grain variation, measured by the standard deviation, is comparable for both the Cu^2+^/Cu and Cu/Cu^2+^ processes (Fig. 3c).

As stated above, *τ* depends on the amount of Cu^2+^ released into the solution during the OCP step (*t* < 0, Fig. 2b), therefore the analysis presented in Fig. 5b and d (extracted from Movie S2 and S3†) can give an insight on the relationship between the two half reactions contributing to Cu corrosion [*i.e.*, Cu oxidation and ORR, eqn (1) and (4)]. The variation in *τ* within a single grain is generally relatively high, for example, comparing the *τ* distributions of grains γ, δ and ε (inset in Fig. 5d), differences in the maximum value of the three distributions are evident (*e.g.*, β has the longest transition time), but the distributions overlap more than in the equivalent *E*_surf_ distributions (inset in Fig. 5d). Nevertheless, due to the large number of *E*_surf_–*t* measurements made in each individual grain, differences in the mean *τ* values are readily distinguished, with *τ*_γ_ = 1.101 s (st. dev. = 0.059 s), *τ*_δ_ = 1.197 s (st. dev. = 0.097 s) and *τ*_ε_ = 1.138 s (st. dev. = 0.096 s).

More broadly, Fig. 5d presents very clear trends in *τ* with crystallographic orientation, evident by considering the same five distinct areas (named A to E), discussed above. Area B, in particular, is the one with the smallest *τ* values (*τ* ≤ 1 s), corresponding to the lowest amount of Cu^2+^ released at OCP, which is consistent with the energetically unfavourable Cu/Cu^2+^ (*i.e.*, most positive values of *E*_surf_) and ORR (*i.e.*, most negative values of *E*_surf_) processes on these planes (consult Fig. 3d and 4d, respectively). In contrast, area E possesses relatively large *τ* values (*τ* ≥ 1.2 s), which may be explained by the relatively favourable Cu/Cu^2+^ process on these planes (although the ORR remains relatively unfavourable). Area C also possesses relatively large *τ* values (*τ* ≥ 1.1 s), which may be the result of a favourable ORR that overcomes the moderately unfavourable Cu oxidation. Area D, where ORR is unfavourable but the Cu/Cu^2+^ is moderately favourable, possesses intermediate *τ* values (*τ* ≈ 1.1 s). The response in area A is more complex, possessing relatively variable *τ* values, *e.g.*, grains closer to the {001}//ND orientation possess *τ* ≥ 1.2 s (*c.f.* area E), while those with higher *C*_1_ values (≥25°) possess *τ* ≤ 1 s (*c.f.* area B). Nevertheless, area A is largely an exception, and the relative ease of the two half reactions, Cu/Cu^2+^ and ORR (from *E*_surf_), is a predictor of the amount of Cu^2+^ released during corrosion at OCP (from *τ*) on the grains situated in areas B–E.

### Grain-dependent open circuit potential step

**Fig. 6:**
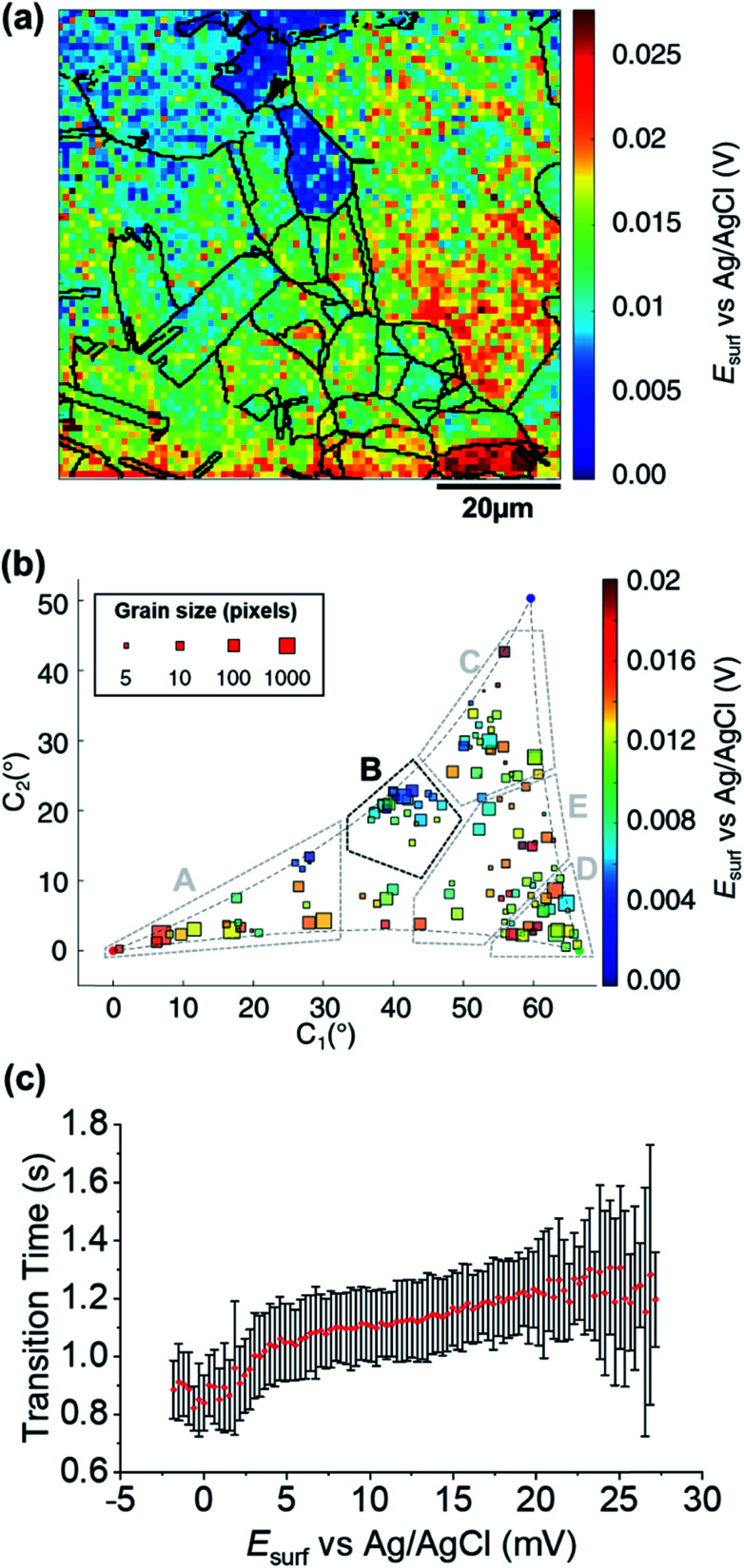
(a) SECCM *E*_surf_ map at OCP on a polycrystalline Cu foil electrode immersed in dodecane. The nanopipette probe contained aerated 10 mM H_2_SO_4_ and contacted an area of 6.4 × 10^−9^ cm^2^ at each location in the map. The map was extracted at time −0.01 s of Movie S2† (negative times are used for indicating the pre-cathodic OCP step). The grain boundaries from Fig. 4b are overlapped on the map. (b) Full grain orientation correlation analysis of *E*_surf_ at −0.01 s from (a) and Fig. S10e†*versus* the average grain orientation, extracted from Fig. 4b and S10b.† Details of the data extracted for each single grain can be found in the ESI, Section S.7 (Fig. S11 and Table S3†). Relevant orientation areas are marked on the graph. (c) Plot of the general correlation between *τ*_Cu^2+^/Cu_ from Fig. 5b and S10d,†*versus* the *E*_surf_ in the OCP step for each point measurement (from Fig. 6a and S10e†). *τ* is represented as the median values of *τ* for each recorded value of OCP (with a step size of 0.31 mV, corresponding to the sensitivity of the measurement), with an error bar representing the standard deviation for the same set of data. Only the OCP points with at least 10 *τ* values are represented.

A map of the OCP extracted from Movie S2† (0.01 s before the application of the cathodic pulse) is shown in Fig. 6a. It is clear that the OCP is grain dependent, as shown in the OCP/crystallographic orientation correlation plot, presented in Fig. 6b. Most clearly by visual inspection, area B, which shows the most negative OCP values (with a median value *E*_OCP,B_ = 0.0046 V, compared to an overall median of 0.0116 V), also have the smallest *τ* values, corresponding to the least amount of Cu^2+^ released in the OCP step (*vide supra*), indicating that the least anodic OCP corresponds to a surface with the highest corrosion resistance. Although the deduction of corrosion susceptibility from OCP measurements may be complicated due to the different surface structural dependences of the kinetics and thermodynamics of the Cu/Cu^2+^ and ORR processes, and the involvement of O_2_ in the Cu/Cu^2+^ process, there is a positive relationship between *τ* (from Fig. 5b and S10d†) and the OCP (from Fig. 6b and S10e†), as shown in Fig. 6c. Thus, while there maybe outliers (note the error bars on *τ* in Fig. 6c), nanoscale OCP measurements provide a guide to metal corrosion susceptibility/resistance, particularly if large datasets can be collected (*i.e.*, on the order of a few thousand points, Fig. 6).

### Probing electrochemical activity at microscopic surface defects

Physical deformations, in particular scratches, are unavoidably introduced during the manufacturing and the finishing (polishing) processes of any metal surface. It is accepted that, in most cases, a macroscopic scratch will possess increased corrosion susceptibility compared to the rest of the metallic surface, predominantly due to induced micro-fractures in the material that act as initiation sites for corrosion-related processes.^81,82^ Although the corrosion-action of several different kinds of physical deformation have been the subject of macroscopic electrochemical investigation and surface analysis techniques,^83–85^ the role of microscopic defects, such as scratches introduced by fine polishing, is still largely unknown. SECCM enables electrochemical activity to be probed at a commensurate scale to microscopic surface defects.

**Fig. 7:**
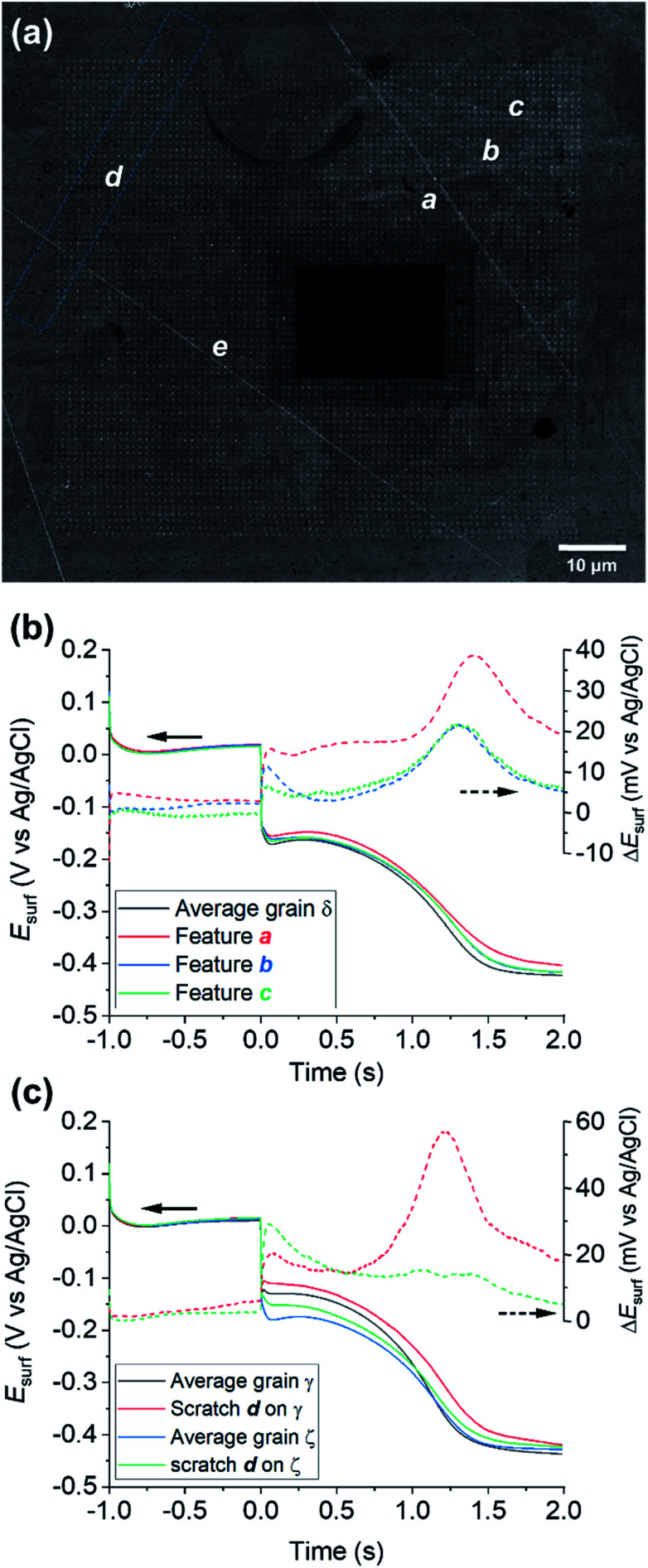
(a) SEM image of the scanned area of Fig. 4 and 5 (Movie S2†), with main surface scratches labelled with the letters a–e. (b–c) Comparison of the average chronopotentiometric (*E*–*t*) curves recorded on the scratches to the surrounding grain(s). In (b), scratches a, b and c are compared to grain δ and in (c), scratch d is compared to grains γ and ζ, which are identified on the corresponding EBSD image, Fig. 4b. Also shown are plots of the difference between the *E*_surf_–*t* curves recorded on the scratches and the surrounding grain, Δ*E*_surf_.

A number of microscopic scratches were observed in the mirror-finished Cu surface employed in this work, as indicated by the SEM and EBSD image shown in Fig. 7a and ESI, Fig. S12,† respectively. Five major scratches can be identified within the cathodic scan area shown in Fig. 4 and 5 and labelled a to e in Fig. 7a. Additionally, at least other 6 features can be identified in the IPFz map (ESI, Fig. S12a†) and *ca.* 20 minor defects can be identified if the disorientation colour map is analysed (ESI, Section S12b†). The discussion here focuses mainly on the features identified under cathodic polarisation, but a few scratches were also identified within anodic polarisation, identified and briefly discussed in the ESI, Section S.8 (Fig. S13†).

Electrochemical analysis of these scratches, achieved by correlating the SECCM and SEM/EBSD maps, reveals that microscopic surface deformations often have a pronounced effect on the (cathodic) surface activity. As an example, Fig. 7b compares the *E*_surf_–*t* pulses obtained from scratches a, b and c of Fig. 7a to the average *E*_surf_–*t* from the surrounding grain, in which they are located (*i.e.*, grain δ in Fig. 5c). Evidently, all three features exhibit enhanced cathodic activity compared to the grain average, with feature a being the most active. It is important to note that the area probed by the nanopipette (≈900 nm diameter for the cathodic pulse, Fig. S2†) is much larger than the topographical deformation (≈50 nm width, as can be seen from the SEM image, Fig. 7a; commensurate with the size of the polishing particles; it can be assumed the depth of the scratch would be similar). Notably, while the scratch is a minor component of the probed area, there is evidently considerably more Cu^2+^ released at OCP in these locations, lengthening the transition time between the Cu^2+^/Cu and ORR processes.

A fascinating aspect of this analysis is that the activity of the scratch seems to be highly dependent on the grain in which it is located. As an example, the results over feature d are analysed in Fig. 7c. The section of scratch d that is located on grain γ behaves very similarly to that of scratch a on grain δ (*i.e.*, Δ*E*_surf_ ≈ +20 mV for both the cathodic processes and a longer transition between the Cu/Cu^2+^ and ORR plateaus). Conversely, on the section of scratch d that is located on grain ζ, Δ*E*_surf_ for the Cu^2+^/Cu process is ≈ +20 mV, compared to ≈0 mV for the ORR. In addition, despite the very similar orientation of grains δ and ζ, the two scratches located on them, a and d, exhibit a completely different behaviour, shown in Fig. 7b and c, respectively. Similar observations can also be made for the several scratches, besides d, found on grain γ, as shown in the ESI, Fig. S12c.† It should also be noted that some surface defects have no apparent effect on *E*_surf_, such as scratch e in Fig. 7a. Evidently, the electrochemical activity induced by surface deformation must depend on the orientation of the underlying grain that is exposed within the defect, in addition to other factors such as the increased surface area resulting from surface roughening, and/or the increased surface strain.

Interestingly, while such variety of enhanced activity is observed for mechanically induced defects in the structure, the same is not generally observed, in the present conditions, for the grain boundaries, as can be seen from the reproduction of the anodic and cathodic *E*_surf_ maps without the boundaries overlaid (ESI, Fig. S14a–c†). It has previously been shown that SECCM possesses sufficient sensitivity to detect the activity of atomic scale defects such as step edges,^45^ grain boundaries^23,27^ and point defects,^86^ with the caveat that these sites need to be highly active (*ca.* orders-of-magnitude) compared to the surrounding basal surfaces, in order to be detected.^87^ While the lack of electrochemical contrast at grain boundaries may indicate that they do not serve as anodic/cathodic sites in the present case, the large size of the droplet footprint (800–900 nm diameter) relative to the grain boundary (*i.e.*, the majority of the measurement area is grain rather than grain boundary) needs to be noted. To better target grain boundaries, an analogous chronopotentiometric SECCM experiment was carried out with a smaller nanopipette probe (*ca.* 150 nm diameter) and higher spatial resolution (250 nm, shown in the ESI, Fig. S14d and e†), but the grain boundary activity still appears to be roughly the average of the two adjacent grains. Thus, while enhanced anodic/cathodic activity at the grain boundary terminations cannot be totally ruled out at this lateral resolution, it is concluded that these sites are not orders-of-magnitude more active than the surrounding grains.

Nevertheless, these results still clearly demonstrate that the microscopic features can play an important role as initiation sites for corrosion-related processes, and further underline the great power of SECCM for identifying and characterising them. Further analysis of scratches and defects by SECCM could be very valuable towards developing a holistic view of metal reactivity and corrosion.

## Conclusions

SECCM was successfully adapted to map grain-dependent electrochemical activity at an aqueous nanodroplet/oil/metal three-phase interface, emulating the conditions encountered during corrosion in industrial/automotive environments, and opening up new prospects for fundamental electrochemical studies at the (sub)microscale. Application of a correlative multi-microscopy approach, in which electrochemistry at a polycrystalline copper surface, from SECCM, was combined with co-located structural information from EBSD, allowed full elaboration of the structure–activity relationship for three corrosion-related processes and their intercorrelation: Cu/Cu^2+^, Cu^2+^/Cu and ORR, and the combination of Cu/Cu^2+^ and ORR during a pre-equilibrium OCP (mixed potential) step. This in-depth analysis method has revealed unique, process-dependent patterns of reactivity for the full spectrum of surface crystallographic orientations, for which the electrochemical activity of high-index surface facets cannot be predicted simply through combination of the {001}, {011} and {111} response(s). Within the full spectrum of surface orientations, it was possible to identify those where the two reactions contributing to Cu corrosion (*i.e.*, Cu/Cu^2+^ and ORR) are most favoured. Nanoscale OCP (mixed potential) measures were furtherly introduced, outlying a procedure to detect Cu^2+^ released into solution at OCP as a marker of the extent of corrosion, as reflected in the chronopotentiometric transition time. This analysis highlights the value of both kinetic mapping and OCP measurements for revealing local corrosion susceptibility with SECCM.

In addition, it was also possible to probe the electrochemical activity of individual (micro)scratches, revealing that these nanoscopic surface defects can serve as cathodic “hot-spots” for corrosion-related processes. Although this work predominantly focused on the behaviour of single grains, the local interplay between corrosion sites (*e.g.*, situated on different grains or grain boundaries), and ultimately the effect of galvanic coupling between them would be amenable to study through the implementation of a “multi-scale” SECCM approach (*i.e.*, with probes of graded diameters). Overall, this work sets a new standard for surface structure–activity studies in (electro)materials and corrosion science, and additionally provides a new configuration for the study of multiphase environments at the nanoscale.

## Conflicts of interest

There are no conflicts to declare.

## Supplementary Material

SC-012-D0SC06516A-s001

SC-012-D0SC06516A-s002

SC-012-D0SC06516A-s003

SC-012-D0SC06516A-s004

SC-012-D0SC06516A-s005
