# Supplementary material for: Nanoscale electrochemistry in a copper/aqueous/oil three-phase system: surface structure–activity-corrosion potential relationships
Source: Chem Sci. 2020 Dec 22;12(8):3055–69. doi: 10.1039/d0sc06516a (PMC8179364; doi:10.1039/d0sc06516a)
Supplement: SC-012-D0SC06516A-s002 [file SC-012-D0SC06516A-s002.pdf]

Electronic Supplementary Material (ESI) for Chemical Science.
This journal is © The Royal Society of Chemistry 2021

Supplementary information for

Nanoscale Electrochemistry in a Copper/Aqueous/Oil Three-phase System: Surface Structure-Activity-Corrosion Potential Relationships

Enrico Daviddi,[1] *Viacheslav Shkirskiy,*[1] *Paul M. Kirkman,*[2] *Mathew P. Robin,*[2] *Cameron L. Bentley*[1,*] *and Patrick R. Unwin*[1,*]

[1] Department of Chemistry, University of Warwick, Coventry CV4 7AL, UK
[2] Lubrizol LTD, Nether Ln, Hazelwood DE56 4AN, UK

Contents

S.1. Tip pulling parameters 3

S.2. Additional SEM images of SECCM scan areas 3

S.3. Finite element method (FEM) simulations of oxygen transport across the oil-water interface. 6

S.4. Movie captions 11

S.5. OCP measurement for Movie S1 12

S.6. Development and details of the 2-dimensional (2D) projections of crystallographic orientation relative to the low-index orientations 12

S.7. Crystallographic details of the grains scanned by SECCM and additional Structure-activity maps 17

S.8. Structure-electrochemistry analysis of surface defects: cathodic and anodic processes 26

References 29

S.1. Tip pulling parameters

For ≈ 400-600nm tips

line 1: HEAT 800, FIL 4, VEL 40, DEL 130, PUL 30;

line 2: HEAT 750, FIL 3, VEL 20, DEL 130, PUL 100.

S.2. Additional SEM images of SECCM scan areas

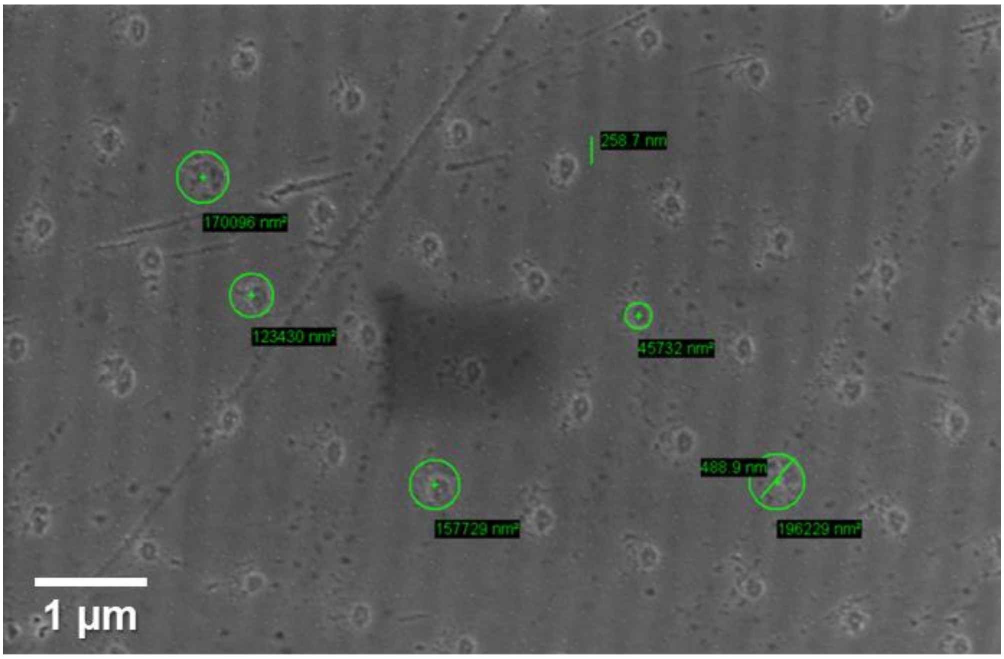

Figure S1. SEM images of the droplet "footprint" residue remaining on the polycrystalline Cu surface after the chronopotentiometric anodic pulse (Movie S1).

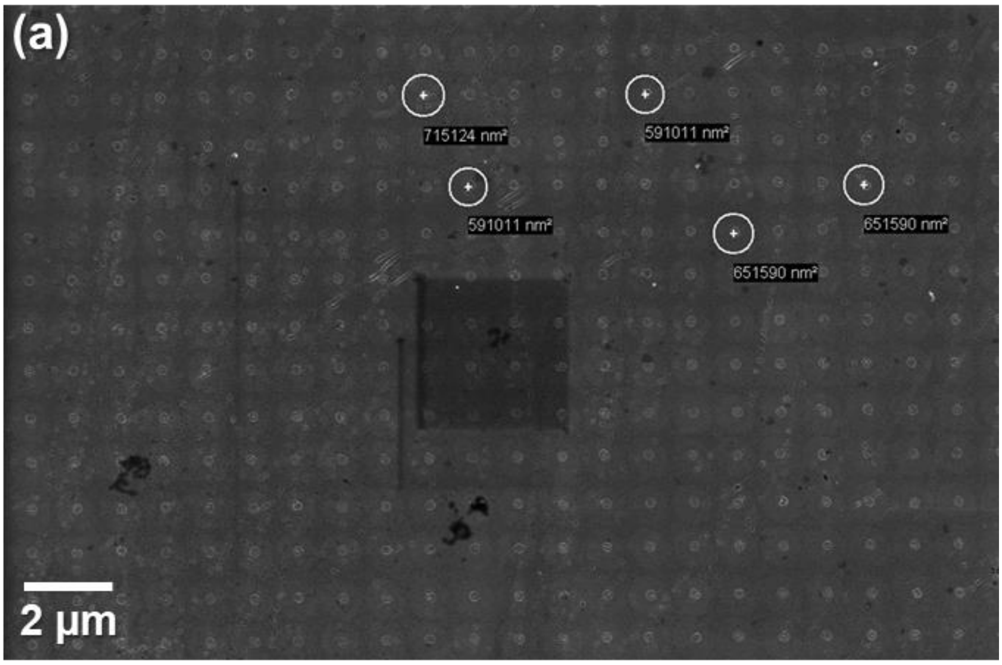

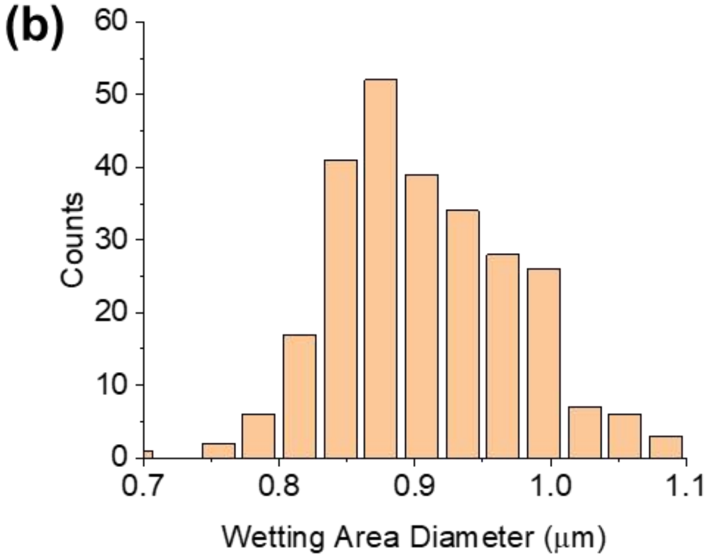

Figure S2. (a) SEM images of the droplet "footprint" residue remaining on the polycrystalline Cu surface after the chronopotentiometric cathodic pulse (Movie S2).(b) Statistical analysis of the wetted area of the droplet meniscus extracted from (a). The average value of the diameter is 0.9 μm, with a standard deviation of 0.16 μm. No significant variation of the wetted area was observed within the scanned area.

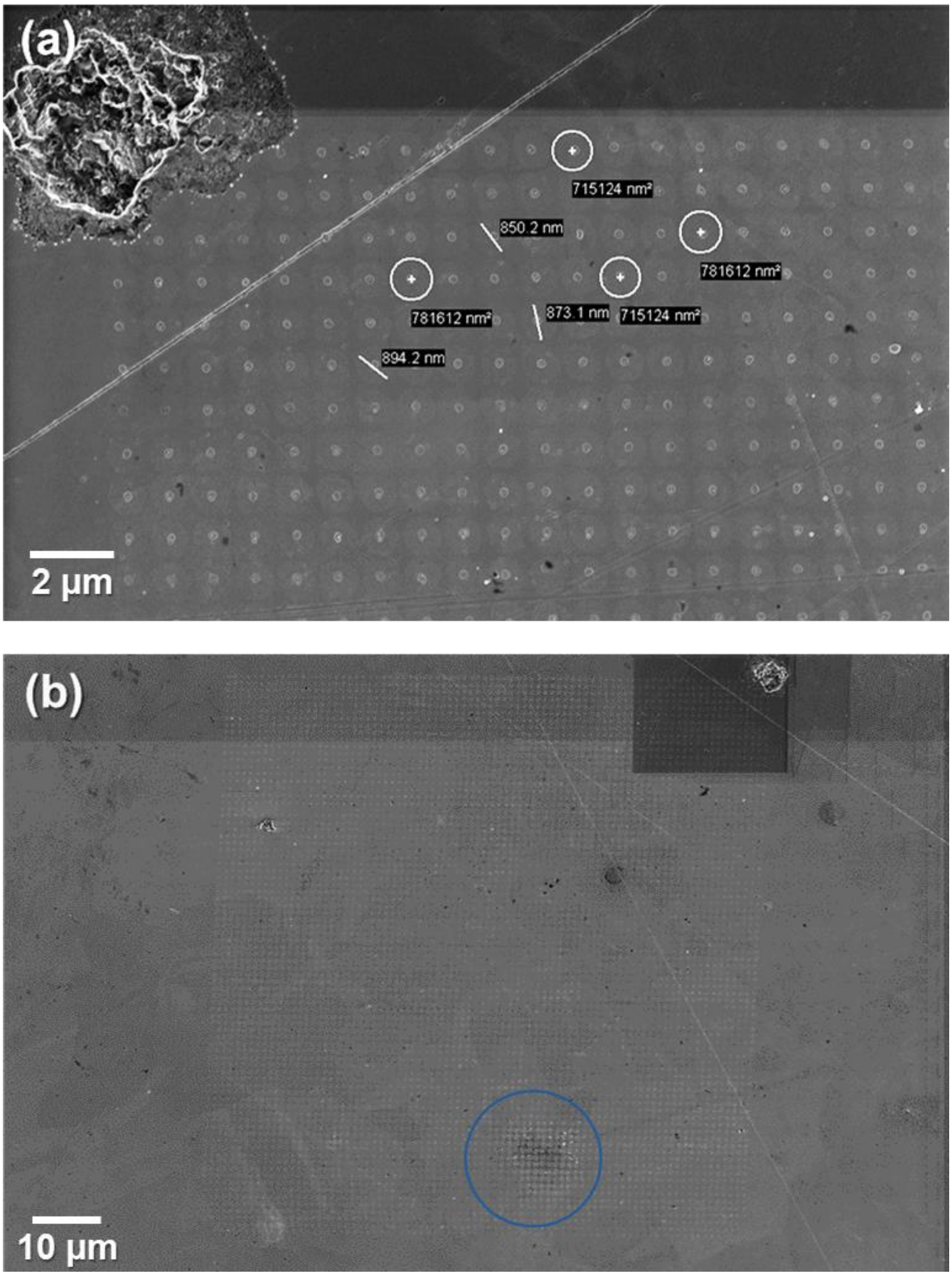

Figure S3. SEM images of the droplet "footprint" residue remaining on the polycrystalline Cu surface after the chronopotentiometric cathodic pulse (Movie S3) at magnifications of **(a)** 5150× and **(b)** 835×. In (b), it is possible to note the contaminated are of the surface that was discarded during grain analysis (highlighted with a circle).

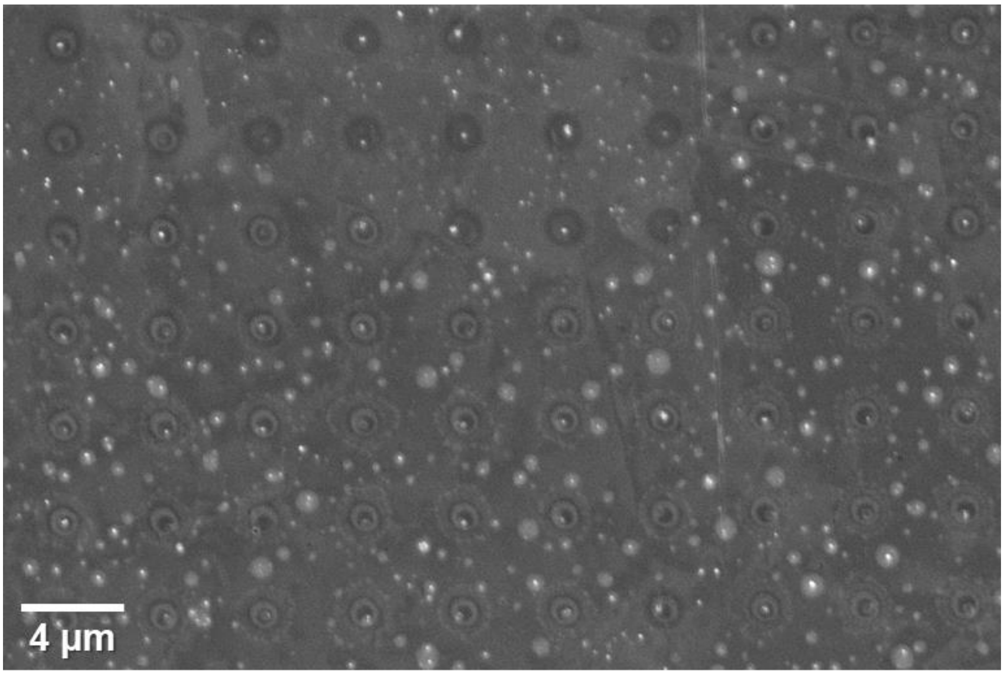

Figure S4. SEM images of the droplet “footprint” residue remaining on the polycrystalline Cu surface after the chronopotentiometric anodic pulse in de-aerated atmosphere. The area used for the calculation of the average wetted area is the wider area of each meniscus trace (the third crown around each point). The average wetted area measured from the present image is $3.16 \cdot 10^{-8}$ cm^2.

S.3. Finite element method (FEM) simulations of oxygen transport across the oil-water interface.

Methods. Finite element method (FEM) simulations were carried out with the COMSOL Multiphysics 5.4 software suite, to simulate the reduction of O_2 to H_2O in an SECCM environment under a steady-state diffusion-limited regime.[1] The simulations considered: |1| the meniscus cell is completely isolated from the surrounding environment and |2| the meniscus cell is in contact with a layer of dodecane with which the reactant (O_2) is exchanged. The general geometry is shown in Figure S5, with the dodecane layer of condition |2| highlighted in orange. In both cases, a single-channel pipette was simulated, with a 2D axisymmetric cylindrical geometry. The bottom boundary (labelled $\boldsymbol{r}_{\mathbf{dr}}$ in Figure S5) represents the working electrode, *i.e.*, the contact area between the nanodroplet meniscus and the Cu substrate. A flux (J) was imposed on this boundary:

$$J_{O_2}^{et} = -k_f [O_2] \quad (S1)$$

with k_f =1.5·10^3 cm s^{-1} to ensure the reaction was diffusion-controlled.

Mass-transport in all the domains was modelled using the transport of diluted species module in COMSOL, with the mass-transport of O_2 assumed to be governed solely by diffusion. Phase-specific diffusion coefficients (D_{ph}) and bulk concentrations (C_{ph}), where *ph* is aqueous (aq) or dodecane (dodec), were employed in the aqueous phase (*i.e.*, within the nanopipette probe and meniscus) and the dodecane phase (where simulated): D_{aq} = 2·10^{-5} cm^2 s^{-1} (ref 2), D_{dodec} = 4.11·10^{-5} cm^2 s^{-1} (ref 3), C_{aq} = 0.26 mM (Ref 4) and C_{dodec} = 2.02 mM, for aerated solution.[4] The pertinent initial concentrations were imposed as boundary conditions both at the top of the tip (labelled $\boldsymbol{r_p}$ in Figure S5) and at the top of the dodecane layer (labelled $\boldsymbol{r_{air}}$ in Figure S5).

In condition |2|, an equilibrium partition flux was imposed at the boundary between the aqueous solution and the dodecane layer (labelled $\boldsymbol{X_{int}}$ in Figure S5):

$$J_{O_2,dodec}^{int} = -k_{in} [O_2]_{dodec} + k_{out} [O_2]_{aq} \quad (S2)$$

$$J_{O_2,aq}^{int} = +k_{in} [O_2]_{dodec} - k_{out} [O_2]_{aq} \quad (S3)$$

with

$$K_{part} = \frac{k_{out}}{k_{in}} \quad (S4)$$

where K_{part} is the partition coefficient between the two phases. In this simulation K_{part} = 7.8 (ref 5) while k_{out} was set to be a high value (k_{out} = 10 cm s^{-1}), such that the system could be considered at equilibrium on the time scale of the calculation. This is reasonable as O_2 transfer across immiscible liquid boundaries is diffusion-limited.[6] In condition |1|, this boundary was set to be a no-flux boundary, to demonstrated that O_2 flux down the nanopipette barrel is

negligible compared to the flux across the oil-water boundary. In addition, unless otherwise stated, the no-flux condition was imposed on all boundaries. The system was solved with stationary condition, adopting the PARDISO solver.[7] The current (i) at the electrode (labelled r_{dr} in Figure S5) was calculated by integration of the O_2 flux through the boundary, assuming a 2 electrons reduction reaction (n = 2):[8]

$$i = \int_0^{2\pi} r d\phi \int_0^{r_{dr}} nFD_{aq} \frac{\partial [O_2]}{\partial z} dr \tag{S5}$$

with r, z and ϕ being the cylindrical coordinates represented in Figure S5.

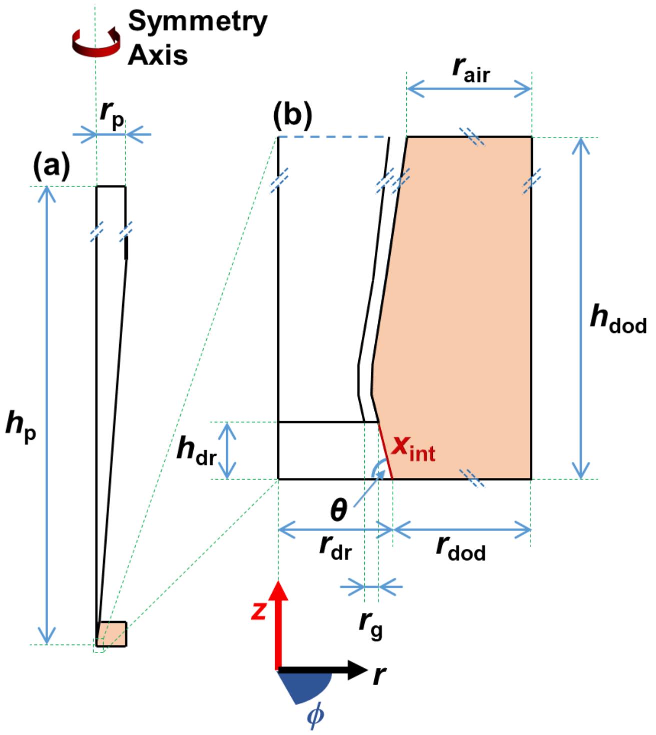

Figure S5. Geometry of the 2D axisymmetric FEM simulations, with (a) defining the entire geometry and (b) a magnification of the nanodroplet (meniscus contact) region. When explicitly considered in the simulations, the dodecane layer is represented by the orange-shaded area. $\boldsymbol{h_p}$ = 5 · 10^{-2} m, $\boldsymbol{r_p}$ = 3.5 · 10^{-4} m, $\boldsymbol{h_{dr}}$ = 200 nm, $\boldsymbol{r_{dr}}$ = 400 nm, $\boldsymbol{r_g}$ = 50 nm, θ = 87°, $\boldsymbol{r_{dod}}$ + $\boldsymbol{r_{dr}}$ = 3 · 10^{-4} m, $\boldsymbol{h_{dod}}$ = 3 · 10^{-4} m, $\boldsymbol{r_{air}}$ = 2.77 · 10^{-4} m. x_{int} is either a no-flux boundary (condition |1|) or the boundary representing the interface between aqueous solution and dodecane (condition |2|).

Results and Discussion. As addressed in previous studies,[1, 9, 10] the configuration of SECCM mimics a gas diffusion electrode, to some extent, with an enhanced flux of gaseous reactants/products across the meniscus-cell (*i.e.*, at the three-phase boundary). Thus, before considering the grain-dependence of the ORR on polycrystalline Cu examined in the main text, finite element method (FEM) simulations were carried out to understand the transport of O_2 across the oil-water interface in SECCM (see Figure 1 of the main text for experimental schematic). As outlined above (see Figure S5), the simulations consider two different conditions: |1| O_2 transport limited to only the aqueous phase (*i.e.*, no gas exchange with the surrounding oil phase) and; |2| O_2 transport in both the aqueous and surrounding oil phase (*i.e.*, gas exchange occurs between oil and aqueous phases). Note that condition |1| was explored to determine the relative contributions of mass-transport down the nanopipette barrel vs. across the liquid-liquid (oil-water) phase boundary, to overall O_2 flux. Simulated O_2 concentration profiles, obtained from a diffusion-controlled four-electron process (*e.g.*, the ORR at high overpotentials) are shown in Figure S6.

Under condition |1| (*i.e.*, no gas exchange with the surrounding oil phase), a diffusion-limited current of 7.9 pA (1.6 mA cm^{-2}) was calculated, which is 5 % of that expected at the same sized inlaid disc microelectrode, in agreement with previous reports of mass transport in SECCM.[11-13] In this case (Figure S6a), as the O_2 is depleted exclusively from the reserve in aqueous solution, the diffusion layer extends tens of μm into the probe, with the concentration reaching 90% of the bulk value (C_{O_2} = 0.26 mM) at *ca.* 80 μm from the working electrode surface.

Under condition |2| (*i.e.*, gas exchange occurs between oil and aqueous phases), because the solubility and the diffusion coefficient for O_2 are greater in the oil phase (2.02 mM and $4.11 \cdot 10^{-5}$ cm^2 s^{-1}, respectively) than the aqueous phase, O_2 transport across the oil-water boundary leads to a greatly enhanced flux (Figure S6b), and diffusion-limited current of 423

pA (84 mA cm^{-2}). As such, the diffusion layer is compressed compared to condition |1|, with the concentration reaching 90% of the bulk value at only *ca.* 20 μm into the pipette probe from the working electrode surface. As highlighted by the constant concentration contours within the oil phase (Figure S6b), the O_2 assumes a radial-spherical diffusion profile, with the oil supplying > 98% of the reactant flux to the electrode surface, at diffusion-control.

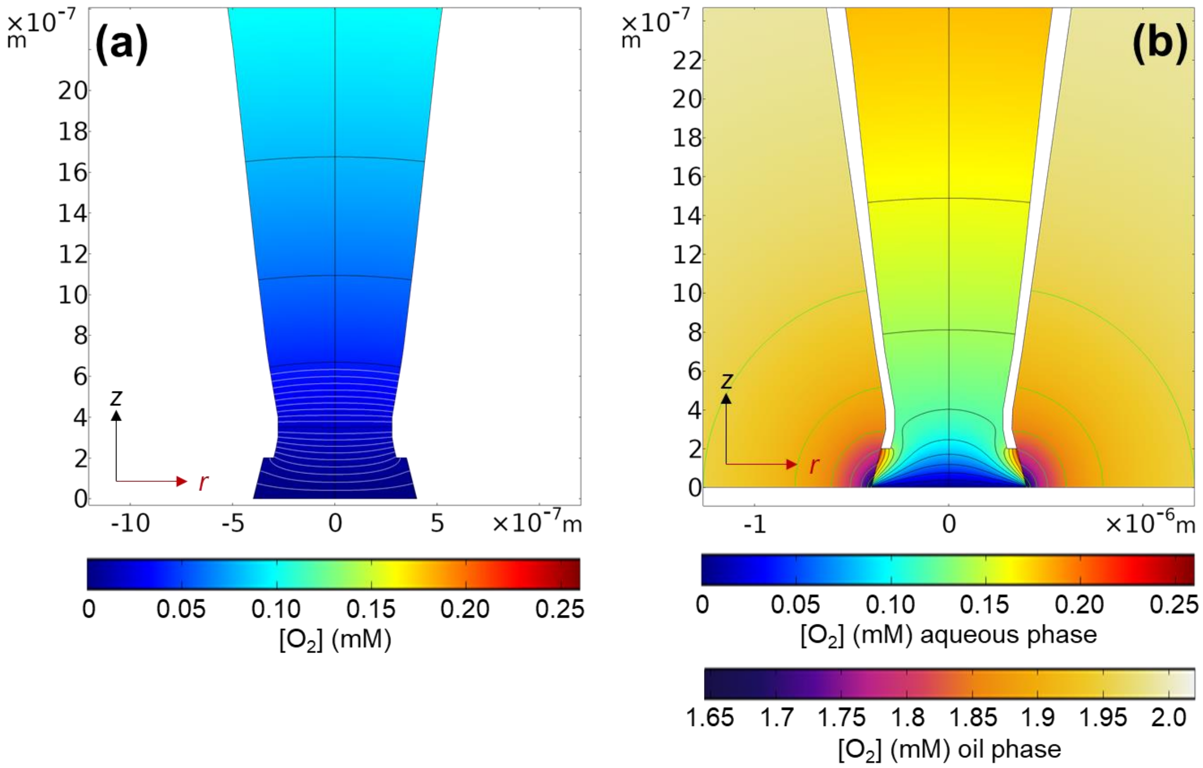

Figure S6.O_2 concentration profiles, taken in the cross section of the nanopipet probe, extracted from the FEM simulations performed with the geometry shown in Figure S5, under steady-state conditions. **(a)** Condition |1|, where O_2 transport is limited to only the aqueous phase (*i.e.*, no gas exchange with the surrounding oil phase). **(b)** Condition |2|, where O_2 transport occurs in both the aqueous and surrounding oil phase (*i.e.*, gas exchange occurs between oil and aqueous phases). In both cases, the green, black and white contours represent increments of 0.05, 0.02 and 0.002 mM, respectively. Note that in (b), the concentrations of O_2 in the aqueous phase and the oil phase are represented with different colour scales and data ranges.

As alluded to above, as I_{app} (0.88 mA cm^{-2}) is small compared the steady-state limiting current (*ca.* 1.6 and 84 mA cm^{-2} under condition |1| and |2|, respectively), so no transition from the ORR to the HER plateau is observed experimentally. Indeed, given the ease with which O_2 can be supplied through a nanodroplet environment, the simulations carried out above

demonstrate the importance of O_2 availability and ORR kinetics for modulating the corrosion-action of acidic nanodroplets.

S.4. Movie captions

Movie S1. Electrochemical surface potential (E_{surf}) movie recorded in the SECCM configuration (127 by 81 pixels, hopping distance 1 μm), obtained by applying an anodic chronopotentiometric pulse to a polycrystalline Cu surface immersed in dodecane. Each pixel represents a single $E-t$ experiment made up of an open circuit potential (OCP, *i.e.*, applied current, i_{app} = 0) step of 1 s (expressed in the movie at negative times, from – 1s to 0) followed by an anodic current pulse of i_{app} = + 10.0 pA (applied current density, I_{app} = + 6 mA cm^{-2}) for 1.5 s. The movie is shown with a time resolution of 0.01 s per frame, with each frame at a given time t_x obtained by averaging E_{surf} between t_x – 0.0015 s and t_x + 0.0015 s. During these measurements, the nanopipet probe (diameter ≈ 400 nm) contained aerated 10 mM H_2SO_4 and contacted an area of 1.6 × 10^{-9} cm^2. The grain boundaries extracted from the main text, Figure 3b were overlapped onto each frame of the video.

Movie S2. Electrochemical surface potential (E_{surf}) movie recorded in the SECCM configuration (77 by 71 pixels, hopping distance 1 μm), obtained by applying a cathodic chronopotentiometric pulse to a polycrystalline Cu surface immersed in dodecane. Each pixel represents a single $E-t$ experiment made up of an OCP (*i.e.*, i_{app} = 0) step of 1 s (expressed in the movie at negative times, from – 1s to 0) followed by a cathodic current pulse of i_{app} = − 5.65 pA (I_{app} = − 0.88 mA cm^{-2}) for 2 s. The movie is shown with a time resolution of 0.01 s per frame, with each frame at a given time t_x obtained by averaging E_{surf} between t_x – 0.0015 s and t_x + 0.0015 s. During these measurements, the nanopipet probe (diameter ≈ 600 nm) contained aerated 10 mM H_2SO_4 and contacted an area of 6.4 × 10^{-9} cm^2. The grain boundaries extracted from the main text, Figure 4c were overlapped onto each frame of the video.

Movie S3. Electrochemical surface potential (E_{surf}) movie recorded in the SECCM configuration (74 by 78 pixels, hopping distance 1 μm), obtained by applying a cathodic chronopotentiometric pulse to a polycrystalline Cu surface immersed in dodecane. Each pixel represents a single $E-t$ experiment made up of an OCP (*i.e.*, i_{app} = 0) step of 1 s (expressed in the movie at negative times, from – 1s to 0) followed by a cathodic current pulse of i_{app} = − 5.65 pA (I_{app} = − 0.88 mA cm^{-2}) for 2 s. The movie is shown with a time resolution of 0.01 s per frame, with each frame at a given time t_x obtained by averaging E_{surf} between t_x – 0.0015 s and t_x + 0.0015 s. During these measurements, the nanopipet probe (diameter ≈ 600 nm) contained aerated 10 mM H_2SO_4 and contacted an area of 6.4 × 10^{-9} cm^2. The grain boundaries extracted from the Supporting Information, Figure S10b were overlapped onto each frame of the video.

S.5. OCP measurement for Movie S1

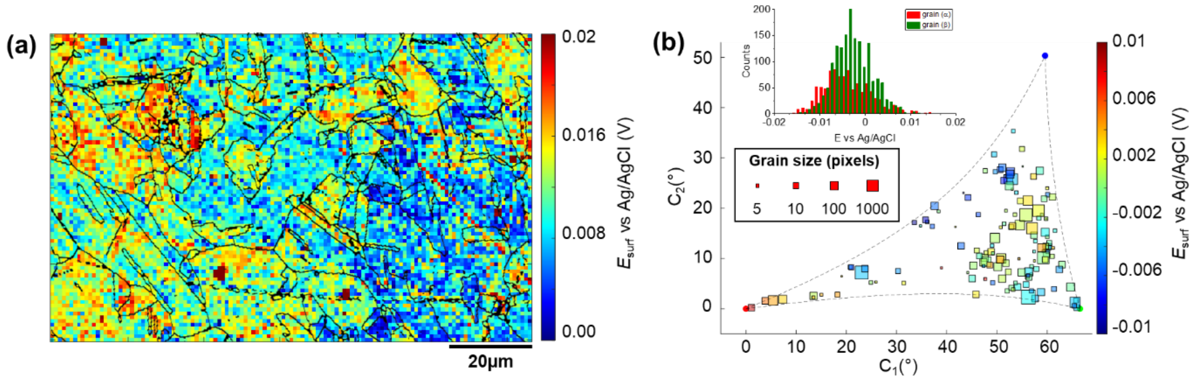

Figure S7. **(a)** SECCM E_{surf} map at OCP (named E_{OCP}) on a polycrystalline Cu foil electrode immersed in dodecane. The nanopipette probe contained aerated 10 mM H_2SO_4 and contacted an area of 1.6×10^{-9} cm^2 at each location in the map. The map was extracted at time − 0.01 s of Movie S1 (negative times are used for indicating the pre-anodic OCP step). The grain boundaries from Figure3b of the main text are overlapped on the map. **(b)** Full grain orientation correlation analysis of E_{OCP} from (a) versus the average grain orientation, extracted from Figure3b, main text. Details on the calculation of the projection coordinates adopted in (b) are discussed in Section S.6, while details of the data extracted for each single grain can be found in Section S.7 (Figure S8 and Table S1). The inset in (b) shows the statistical distribution of E_{OCP} extracted from grains α and β, marked in Figure3b, main text.

S.6. Development and details of the 2-dimensional (2D) projections of crystallographic orientation relative to the low-index orientations

In order to represent the average orientation of each measured grain on a bi-dimensional plane, a new kind of 2D projection was developed herein, allowing the coordinates of each plane to be easily calculated and visualised from the Euler angles. The orientation of a generic plane, α, in space can be defined by three Euler angles, φ_1, Φ and φ_2. From these angles, it is possible to obtain the Miller indices (h,k,l) of the plane parallel to the normal direction (ND):

$$h = n \sin \Phi \sin \varphi_2 \quad \text{(S6)}$$

$$k = n \sin \Phi \cos \varphi_2 \quad \text{(S7)}$$

$$l = n \cos \Phi \quad \text{(S8)}$$

As it can be seen from Eqs (S6) to (S8), the Miller indices depend only on the latter two Euler angles, with φ_1 corresponding to the rotation of the plane relative to ND.

Due to the symmetry of the cubic system to which Cu belongs (crystal group 225), it was assumed that all the families of the plane {*h*,*k*,*l*} had equivalent structures, thus the Miller indexes of planes α (*h*,*k*,*l*) were simply ordered from smallest to largest, so that $h' \le k' \le l'$, with h', k' and l' being the rearranged indexes. This was done in order to obtain comparable orientations for the following steps (*vide infra*). For instance, using this convention, planes (100), (010) and (001) are all taken to be equivalent to (001), sorting (*h*,*k*,*l*) from smallest to largest. Once the Miller indices for each plane α were calculated using Eqs (S6) − (S8), the angle between α and each of the three low-index planes employed for cubic system representation, (001), (011), (111), was calculated, respectively as γ_1, γ_2 and γ_3:

$$\cos\gamma = \frac{h_1'h_2' + k_1'k_2' + l_1'l_2'}{\sqrt{(h_1')^2 + (k_1')^2 + (l_1')^2}\sqrt{(h_2')^2 + (k_2')^2 + (l_2')^2}} \tag{S9}$$

with (h'_1,k'_1,l'_1) being the Miller index of α and (h'_2,k'_2,l'_2) being the Miller indexes of the considered low index plane. Therefore, each plane α could be described respectively by three coordinates; for example, calculated values for each the low-index planes are shown in Table S1. Note that as alluded to above, low-index grains (001), (011) and (111) were chosen to fulfil the requirement of sorting (*h*,*k*,*l*) from smallest to largest (*vide supra*).

Table S1.. Values of γ_1, γ_2 and γ_3 for the low index planes in the cubic system.

Plane	γ_1 001 (˚)	γ_2 011 (˚)	γ_3 111 (˚)
001	0	45	54.736
011	45	0	35.264
111	54.736	35.264	0

The points representing (001), (011) and (111) planes in the γ_1, γ_2, γ_3 space are called P_1, P_2 and P_3. Such coordinate values will be expressed with letters, so that P_1 (*0,a,b*), P_2 (*a,0,c*) and P_3 (*b,c,0*), with $a = 45^\circ$, $b = 54.736^\circ$ and $c = 35.264^\circ$.

By calculating γ_1, γ_2 and γ_3 for each generic plane α of the cubic system of Miller indices (*h*',*k*',*l*') as defined above, it can be shown that all calculated points, P, lay on a hyperbolic plane that passes through P_1, P_2 and P_3. This can be qualitatively explained by considering that the miller indexes (*h,k,l*) are not totally independent, being all calculated from the same two Euler angles (Φ and φ_2). Specifically, from the re-elaboration of Eqs (S6), (S7) and (S8):

$$k = \sqrt{1-l}\left(1-\frac{h^2}{1-l}\right) \text{ with } 0 \le l < 1. \tag{S10}$$

Therefore, a useful and simple 2D representation of the grains is introduced by calculating the projection of each point in the (γ_1, γ_2, γ_3) space on the plane passing through P_1, P_2 and P_3. Such a plane will be represented by the following equation:

$$u\gamma_1 + v\gamma_2 + w\gamma_3 + k = 0, \tag{S11}$$

with:

$$u = ac + bc - c^2 \approx 2273.5382^{\circ^2}, \tag{S12}$$

$$v = cb + ab - b^2 \approx 1397.3333^{\circ^2}, \tag{S13}$$

$$w = ac + ab - a^2 = 2025^{\circ^2} \tag{S14}$$

and

$$k = -2abc = -173719.61^{\circ^3}, \tag{S15}$$

a, *b* and *c* correspond to the angles defined above. In order to represent the points over this plane, two Cartesian coordinates were arbitrary defined as follows: *x* axis as the line passing

through P_1 and P_3, *y* axis as the line passing through P_1 and orthonormal, to *x*. The direction of the axis was defined in order of P_2 having both positive coordinates in this projection. Such coordinates were called C_1 (*x*-axis) and C_2 (*y*-axis). Therefore, C_1 and C_2 can be calculated from γ_1, γ_2, γ_3 for each considered plane. If the following constants are defined:

$$\mu_1 = \sqrt{c^2 + b^2 + 2a^2 - 2bc} \approx 66.55^{\circ} \tag{S16}$$

$$\mu_2 = \sqrt{u^2 + v^2 + w^2} \approx 3349.9465^{\circ^2} \tag{S17}$$

$$\epsilon 1_{\gamma_1} = \frac{a}{\mu_1} \approx 0.6761 \tag{S18}$$

$$\epsilon 1_{\gamma_2} = -\frac{a}{\mu_1} \approx -0.6761 \tag{S19}$$

$$\epsilon 1_{\gamma_3} = \frac{c-b}{\mu_1} \approx -0.2926 \tag{S20}$$

$$\epsilon 2_{\gamma_1} = \frac{w(c-b) + va}{\mu_1 \mu_2} \approx 0.1052 \tag{S21}$$

$$\epsilon 2_{\gamma_2} = \frac{-u(c-b) + va}{\mu_1 \mu_2} \approx 0.4806 \tag{S17}$$

$$\epsilon 2_{\gamma_3} = -\frac{a(u+w)}{\mu_1 \mu_2} \approx -0.8676 \tag{S22}$$

Then for each generic plane, P:

$$C_1^P = \epsilon 1_{\gamma_1}(\gamma_1^P) + \epsilon 1_{\gamma_2}(\gamma_2^P - a) + \epsilon 1_{\gamma_3}(\gamma_3^P - b) \tag{S23}$$

$$C_2^P = \epsilon 2_{\gamma_1}(\gamma_1^P) + \epsilon 2_{\gamma_2}(\gamma_2^P - a) + \epsilon 2_{\gamma_3}(\gamma_3^P - b) \tag{S24}$$

A Matlab script for calculating the two coordinates as per Eqs. S23 and S24, starting from the average Euler angles extracted from a generic EBSD map, is attached to the ESI.

The position of some representative planes in coordinates C_1 and C_2 are shown in Figure S8. On this plot, in this coordinate system, P_1 (0,0), P_2 (66.5517˚,0), P_3 (59.6074˚,50.336˚). All generic planes α (*i.e.*, all grains) lay in a section delimited by the following three lines, as shown in Figure S8: line 1, representing the family of planes with miller indices {m,m,1}, where 0 < m < 1; line 2, representing the family of planes with miller indices {0,n,1}, where 0 < n < 1 and; line 3, representing the family of planes with miller indices {q,1,1}, where 0 < q < 1.

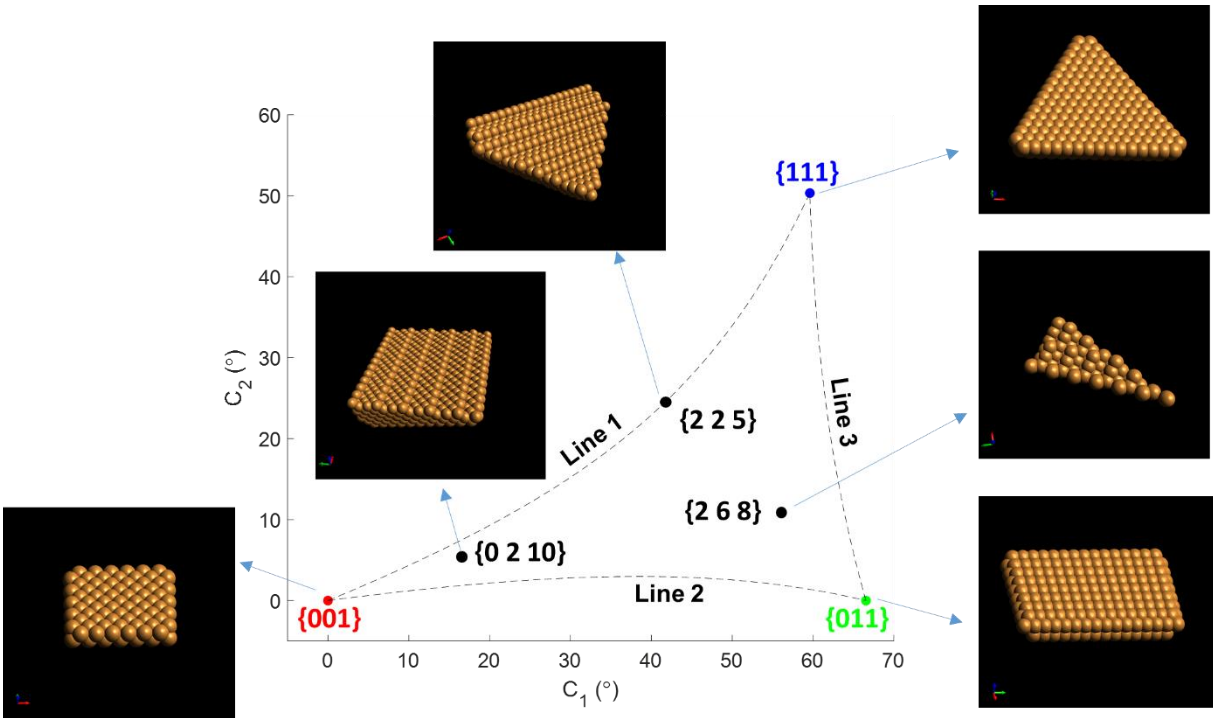

Figure S8. Two-dimensional projection of grain orientations in a fcc cubic crystal system, represented in arbitrary coordinates. The black lines delineate the space that contains all possible grain orientations (given the symmetry of the cubic system). The position of the low-index grains is also highlighted, as well as the surface structure of a few exemplar grains indicated on the plot.

S.7. Crystallographic details of the grains scanned by SECCM and additional Structure-activity maps

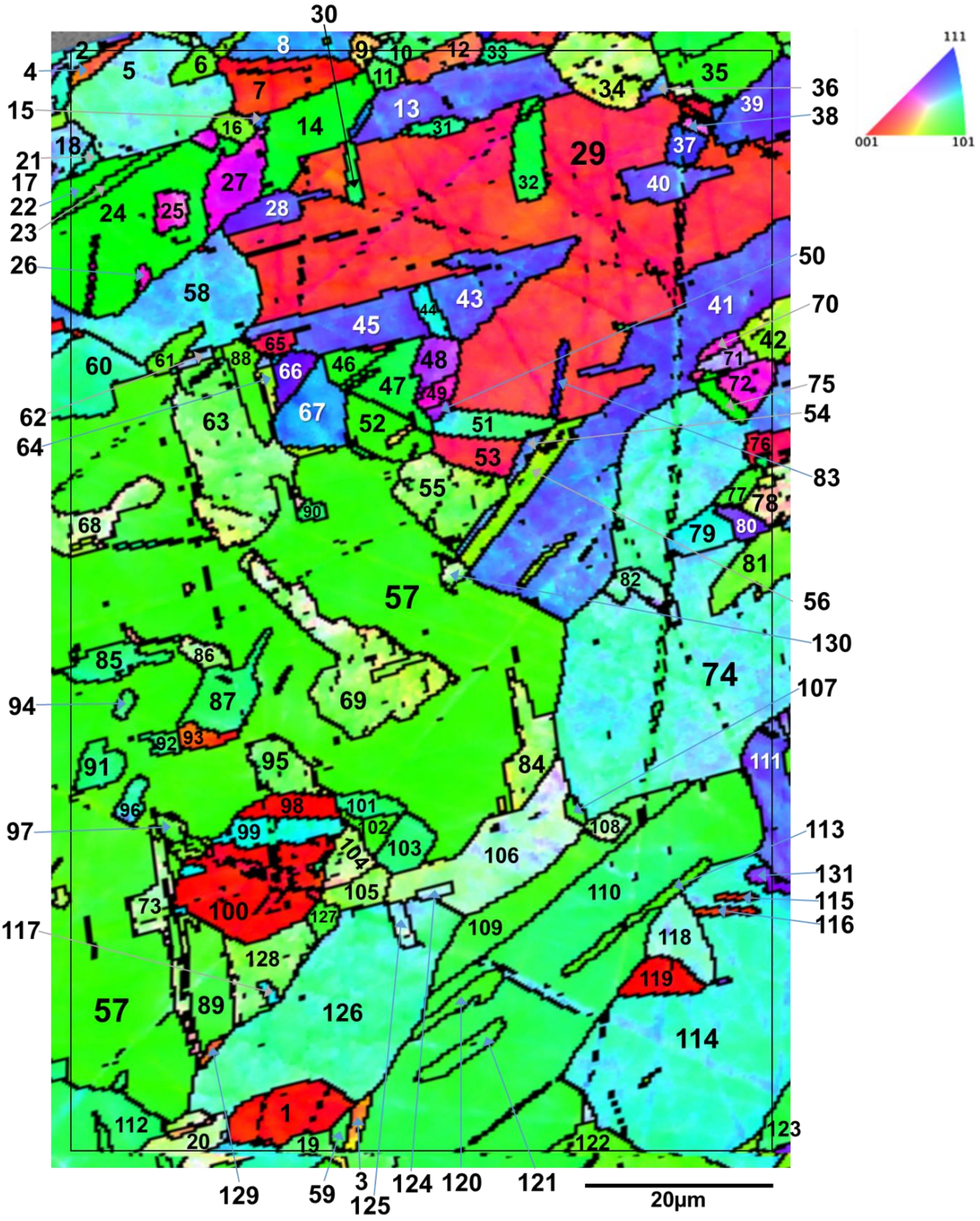

Figure S9. Definition of the grain ID for each grain analysed from Movie S1 (*i.e.*, reproduction of the EBSD data shown in the main text, Figure 3b). The EBSD map was rotated anticlockwise by 90˚ for a clearer visualisation.

Table S2. List of all grains analysed by SECCM (Supporting Information, Movie S1 and main text, Figure 3), with the average Euler angles, Miller indices and Projection Coordinates and E_{surf} listed for each one. The grain IDs correspond to those defined in Figure S9.

ID	Average Euler angles (˚)			Average Miller indices			Projection Coordinates (˚)		Average E_{surf} t = 2s (V vs Ag/AgCl)	Average E_{OCP} t = − 0.01s (V vs Ag/AgCl)	Size (pixels)
	φ_1	Φ	φ_2	h	k	l	C_1	C_2			
1	182.61	4.67	21.64	0.030	0.076	0.997	7.32	1.88	0.0742	0.00611	63
2	257.65	37.98	25.05	0.261	0.558	0.788	53.59	18.71	0.0710	0.00072	7
3	295.68	20.18	9.69	0.058	0.340	0.939	31.43	5.11	0.0664	0.00062	7
4	132.72	15.93	15.22	0.072	0.265	0.962	24.90	5.23	0.0696	0.00057	6
5	297.51	38.16	67.99	0.573	0.232	0.786	54.57	16.64	0.0663	0.00204	146
6	215.84	34.34	87.36	0.564	0.026	0.826	52.06	3.54	0.0695	0.00143	11
7	212.25	8.56	78.45	0.146	0.030	0.989	13.48	2.55	0.0803	0.00107	49
8	88.07	43.61	60.42	0.600	0.340	0.724	57.31	26.33	0.0761	0.00331	20
9	21.15	25.3	74.37	0.412	0.115	0.904	38.96	8.13	0.0700	0.00876	3
10	286.4	39.9	73.26	0.614	0.185	0.767	57.67	13.44	0.0698	− 0.00165	8
11	159.29	38.14	10.85	0.116	0.607	0.787	56.92	8.42	0.0704	0.00158	7
12	124.44	19.62	19.87	0.114	0.316	0.942	30.30	7.50	0.0754	− 0.00442	18
13	97.38	39.37	56.05	0.526	0.354	0.773	52.27	25.81	0.0722	− 0.00778	90
14	166.95	44.24	85.25	0.695	0.058	0.716	64.69	4.31	0.0691	− 0.00183	74
15	90.09	42.47	56.55	0.563	0.372	0.738	55.17	28.32	0.0700	− 0.00004	2
16	219.56	32.18	83.99	0.530	0.056	0.846	49.16	5.18	0.0728	− 0.00376	10
17	260.23	28.44	41.15	0.313	0.359	0.879	38.39	19.37	0.0675	− 0.00045	4
18	256.5	39.7	27.2	0.292	0.568	0.769	54.67	21.44	0.0670	0.00372	23
19	283.6	36.42	13.55	0.139	0.577	0.805	54.40	9.95	0.0636	0.00766	3
20	293.2	30.6	70.45	0.480	0.170	0.861	46.00	11.60	0.0635	− 0.00259	14
21	165.02	42.65	68.64	0.631	0.247	0.736	58.94	18.71	0.0705	− 0.00765	4
22	164.3	44.51	0.14	0.002	0.701	0.713	65.91	0.23	0.0671	− 0.00345	22
23	55.01	44.66	1.83	0.022	0.703	0.711	65.80	1.66	0.0673	− 0.00002	14
24	165.4	44.36	88.58	0.699	0.017	0.715	65.61	1.27	0.0686	− 0.00389	192
25	263.53	26.44	36.95	0.268	0.356	0.895	37.13	16.39	0.0726	− 0.00368	12
26	71.11	25.22	49.64	0.325	0.276	0.905	34.79	16.48	0.0683	− 0.00050	3
27	257.24	28.58	44.33	0.334	0.342	0.878	37.59	20.47	0.0713	− 0.00516	40
28	101.9	39.02	52.37	0.499	0.384	0.777	50.67	27.66	0.0717	− 0.00658	20
29	235.69	15.15	62.07	0.231	0.122	0.965	23.06	7.20	0.0788	− 0.00447	987
30	351.22	42.2	77.86	0.657	0.141	0.741	61.02	10.43	0.0697	0.00199	12
31	61.74	44.39	16.72	0.201	0.670	0.715	61.57	15.52	0.0678	− 0.00325	8
32	349.23	43.73	80.27	0.681	0.117	0.723	62.97	8.78	0.0666	− 0.00101	31
33	61.51	42.36	16.48	0.191	0.646	0.739	60.03	14.30	0.0668	0.00047	6
34	348.35	29.65	13.93	0.119	0.480	0.869	45.33	8.58	0.0689	0.00041	57
35	56.38	39.11	86.76	0.630	0.036	0.776	58.79	3.30	0.0702	− 0.00394	56
36	96.16	39.2	58.97	0.542	0.326	0.775	53.04	23.75	0.0665	0.00169	3
37	316.16	43.64	36.74	0.413	0.553	0.724	55.00	31.79	0.0737	0.00224	13
38	120.96	29.09	58.52	0.415	0.254	0.874	41.73	16.30	0.0755	0.00209	2
39	100.15	39.44	52.41	0.503	0.388	0.772	51.08	28.05	0.0725	− 0.00587	52

40	97.89	38.66	55.52	0.515	0.354	0.781	51.42	25.49	0.0727	− 0.00833	31
41	98	39.93	56.86	0.537	0.351	0.767	53.05	25.79	0.0715	− 0.00427	325
42	204.42	29.34	81.8	0.485	0.070	0.872	45.09	6.00	0.0690	− 0.00022	28
43	98.7	40.23	55	0.529	0.370	0.763	52.70	27.27	0.0722	− 0.00656	71
44	177.42	45.23	23.97	0.288	0.649	0.704	60.13	22.68	0.0697	− 0.00483	12
45	98.95	39.96	55.41	0.529	0.365	0.766	52.60	26.76	0.0717	− 0.00661	67
46	231.04	41.11	4.51	0.052	0.655	0.753	61.37	3.94	0.0695	− 0.00630	15
47	349.61	42.9	81.04	0.672	0.106	0.733	62.45	7.83	0.0648	− 0.00417	36
48	81.87	32.85	38.15	0.335	0.427	0.840	44.26	22.11	0.0692	− 0.00676	21
49	286.85	24.92	46.28	0.305	0.291	0.907	33.56	17.14	0.0713	− 0.00877	10
50	79.65	32.75	40.66	0.352	0.410	0.841	43.33	23.04	0.0749	− 0.01307	2
51	62.12	39.97	18.18	0.200	0.610	0.766	57.38	14.63	0.0646	− 0.00137	29
52	272.1	38.43	0.65	0.007	0.622	0.783	57.62	2.02	0.0672	− 0.00424	36
53	239.02	15.76	59.4	0.234	0.138	0.962	23.71	7.98	0.0772	− 0.00434	28
54	96.52	39.61	60.4	0.554	0.315	0.770	53.87	23.10	0.0667	− 0.00170	6
55	79.58	33.05	77.89	0.533	0.114	0.838	50.19	8.37	0.0646	0.00087	67
56	4.82	30.23	89.91	0.503	0.001	0.864	45.83	2.84	0.0646	0.00214	38
57	200.12	37.45	89.09	0.608	0.010	0.794	56.28	2.32	0.0672	− 0.00305	1654
58	77.88	40.98	63.89	0.589	0.289	0.755	56.16	21.53	0.0639	0.00291	147
59	162.44	38.81	12.17	0.132	0.613	0.779	57.49	9.52	0.0597	0.00306	3
60	117.65	42.21	17.61	0.203	0.640	0.741	59.60	15.22	0.0640	− 0.00267	64
61	343.47	34.84	3.99	0.040	0.570	0.821	52.87	4.14	0.0664	0.00143	13
62	63.11	36.86	62.68	0.533	0.275	0.800	51.80	19.50	0.0655	0.00364	4
63	317.5	33.76	14.67	0.141	0.538	0.831	50.86	9.98	0.0654	0.00214	137
64	321.28	28.76	11.2	0.093	0.472	0.877	44.20	7.19	0.0617	− 0.00025	3
65	247.08	14.28	52	0.194	0.152	0.969	20.80	8.29	0.0799	− 0.00320	11
66	30.47	39.65	47.62	0.471	0.430	0.770	49.45	30.72	0.0702	− 0.00661	16
67	103.19	45.22	29.37	0.348	0.619	0.704	58.57	27.46	0.0687	− 0.00124	52
68	81.77	31.43	73.83	0.501	0.145	0.853	47.57	10.16	0.0642	0.00156	44
69	80.47	31.54	75.44	0.506	0.132	0.852	47.90	9.35	0.0662	− 0.00020	131
70	286.19	26.29	49.87	0.339	0.285	0.897	36.15	17.27	0.0725	− 0.00473	6
71	93.38	32.79	31.01	0.279	0.464	0.841	46.29	18.71	0.0729	− 0.00663	10
72	287.86	26.3	48.55	0.332	0.293	0.896	35.81	17.67	0.0729	− 0.01017	22
73	314.52	32.55	17.64	0.163	0.513	0.843	48.86	11.31	0.0658	− 0.00234	27
74	84.71	39.77	66.57	0.587	0.254	0.769	55.81	18.65	0.0665	0.00113	707
75	23.48	44.14	89.49	0.696	0.006	0.718	65.44	0.58	0.0680	− 0.00417	9
76	254.61	14.37	37.33	0.150	0.197	0.969	21.00	8.25	0.0768	− 0.00518	16
77	133.18	37.21	82.04	0.599	0.084	0.796	56.09	6.35	0.0689	− 0.00076	11
78	327.81	28.68	23.26	0.190	0.441	0.877	42.85	12.50	0.0728	− 0.00684	30
79	357.27	44.18	23.9	0.282	0.637	0.717	59.41	21.90	0.0671	− 0.00121	25
80	277.72	38.24	51.69	0.486	0.384	0.785	49.69	27.25	0.0718	− 0.00376	12
81	349.83	33.49	7.54	0.072	0.547	0.834	51.03	5.97	0.0659	0.00214	45
82	175.77	36.69	22.62	0.230	0.552	0.802	52.85	16.26	0.0643	0.00115	14
83	313.7	43.97	42	0.465	0.516	0.720	53.27	35.36	0.0736	− 0.00305	6
84	317.08	31.12	14.56	0.130	0.500	0.856	47.32	9.25	0.0669	0.00128	47
85	120.23	41.86	14.33	0.165	0.647	0.745	60.15	12.22	0.0649	0.00018	26
86	82.93	31.82	72.25	0.502	0.161	0.850	47.88	11.12	0.0675	0.00258	10

87	277.53	41.28	76.89	0.643	0.150	0.751	59.91	10.96	0.0628	− 0.00091	37
88	203.22	35.7	85.53	0.582	0.045	0.812	54.11	4.33	0.0672	− 0.00203	28
89	207.24	36.42	80.58	0.586	0.097	0.805	54.93	7.24	0.0639	− 0.00355	55
90	120.58	41.61	13.4	0.154	0.646	0.748	60.16	11.32	0.0668	− 0.00561	6
91	278.38	42.11	75.59	0.649	0.167	0.742	60.36	12.39	0.0692	− 0.00401	19
92	121.6	40.84	13.85	0.157	0.635	0.757	59.30	11.44	0.0685	0.00031	5
93	140.87	11.64	83.43	0.200	0.023	0.979	18.23	2.79	0.0761	0.00509	13
94	277.53	39.17	75.53	0.612	0.158	0.775	57.43	11.38	0.0670	− 0.00104	4
95	79.87	34.31	77.09	0.549	0.126	0.826	51.80	9.07	0.0654	0.00148	37
96	283.17	41.88	68.87	0.623	0.241	0.745	58.36	18.06	0.0667	− 0.00075	12
97	76.39	34.47	80.13	0.558	0.097	0.824	52.29	7.31	0.0668	0.00128	19
98	34.21	0.7	79.01	0.012	0.002	1.000	1.11	0.21	0.0791	0.00931	28
99	84.18	46.17	67.2	0.665	0.280	0.693	61.08	22.23	0.0662	0.00204	31
100	84.83	3.49	28.46	0.029	0.054	0.998	5.39	1.63	0.0795	0.00509	130
101	119.36	42.39	15.06	0.175	0.651	0.739	60.44	13.07	0.0662	− 0.00142	16
102	196	37.73	3.35	0.036	0.611	0.791	56.90	3.54	0.0686	− 0.00630	11
103	278.11	41.52	75.24	0.641	0.169	0.749	59.73	12.45	0.0629	0.00097	24
104	82.05	30.75	72.77	0.488	0.151	0.859	46.52	10.49	0.0680	− 0.00009	23
105	280.78	31.94	13.1	0.120	0.515	0.849	48.58	8.69	0.0652	0.00474	22
106	122	33.7	21.59	0.204	0.516	0.832	49.60	14.06	0.0660	0.00103	135
107	10.62	37.86	81.38	0.607	0.092	0.790	56.87	6.83	0.0676	0.00097	4
108	353.44	32.94	19.57	0.182	0.512	0.839	49.04	12.55	0.0703	− 0.00287	8
109	251.53	37.49	80.57	0.600	0.100	0.793	56.31	7.35	0.0682	− 0.00305	84
110	163.45	40.47	12.41	0.139	0.634	0.761	59.26	10.13	0.0633	− 0.00009	497
111	277.79	39.02	57.72	0.532	0.336	0.777	52.48	24.43	0.0683	0.00051	73
112	255.03	40.68	75.25	0.630	0.166	0.758	58.93	12.13	0.0577	− 0.00320	61
113	248.78	36.92	84.54	0.598	0.057	0.799	55.81	4.81	0.0659	− 0.00239	19
114	265.43	42.28	67.44	0.621	0.258	0.740	58.30	19.51	0.0636	0.00240	465
115	102.91	9.56	11.07	0.032	0.163	0.986	15.04	2.79	0.0711	0.00484	5
116	106.64	8.55	8.21	0.021	0.147	0.989	13.44	2.26	0.0767	0.00291	5
117	242.78	45.57	25.15	0.303	0.646	0.700	60.03	23.98	0.0647	0.00128	4
118	123.05	36.96	22.7	0.232	0.555	0.799	53.12	16.46	0.0634	0.00298	34
119	67.88	2.64	46.56	0.033	0.032	0.999	3.83	1.57	0.0778	0.00703	27
120	250.35	37.44	82.21	0.602	0.082	0.794	56.41	6.25	0.0630	− 0.00050	20
121	251.81	38.82	79.99	0.617	0.109	0.779	57.87	7.92	0.0635	− 0.00030	15
122	8.13	35.13	3.32	0.033	0.574	0.818	53.24	3.79	0.0641	0.00054	6
123	315.33	40.73	11.88	0.134	0.639	0.758	59.65	9.77	0.0641	0.00351	20
124	230.3	35.11	66.12	0.526	0.233	0.818	50.77	16.19	0.0658	0.00199	4
125	229.38	35.81	65.62	0.533	0.242	0.811	51.44	16.92	0.0681	0.00016	7
126	319.84	40.02	19.87	0.219	0.605	0.766	57.00	16.00	0.0631	0.00357	295
127	206.16	37.33	81.7	0.600	0.088	0.795	56.22	6.58	0.0703	− 0.00630	7
128	317.33	33.59	14.39	0.137	0.536	0.833	50.67	9.78	0.0624	0.00507	46
129	124.88	16.58	75.4	0.276	0.072	0.958	25.91	5.31	0.0675	0.00382	3
130	84.72	32.75	72.96	0.517	0.159	0.841	49.21	11.05	0.0653	0.00341	5
131	341.09	37.15	40.94	0.396	0.456	0.797	47.68	27.44	0.0657	0.00519	11

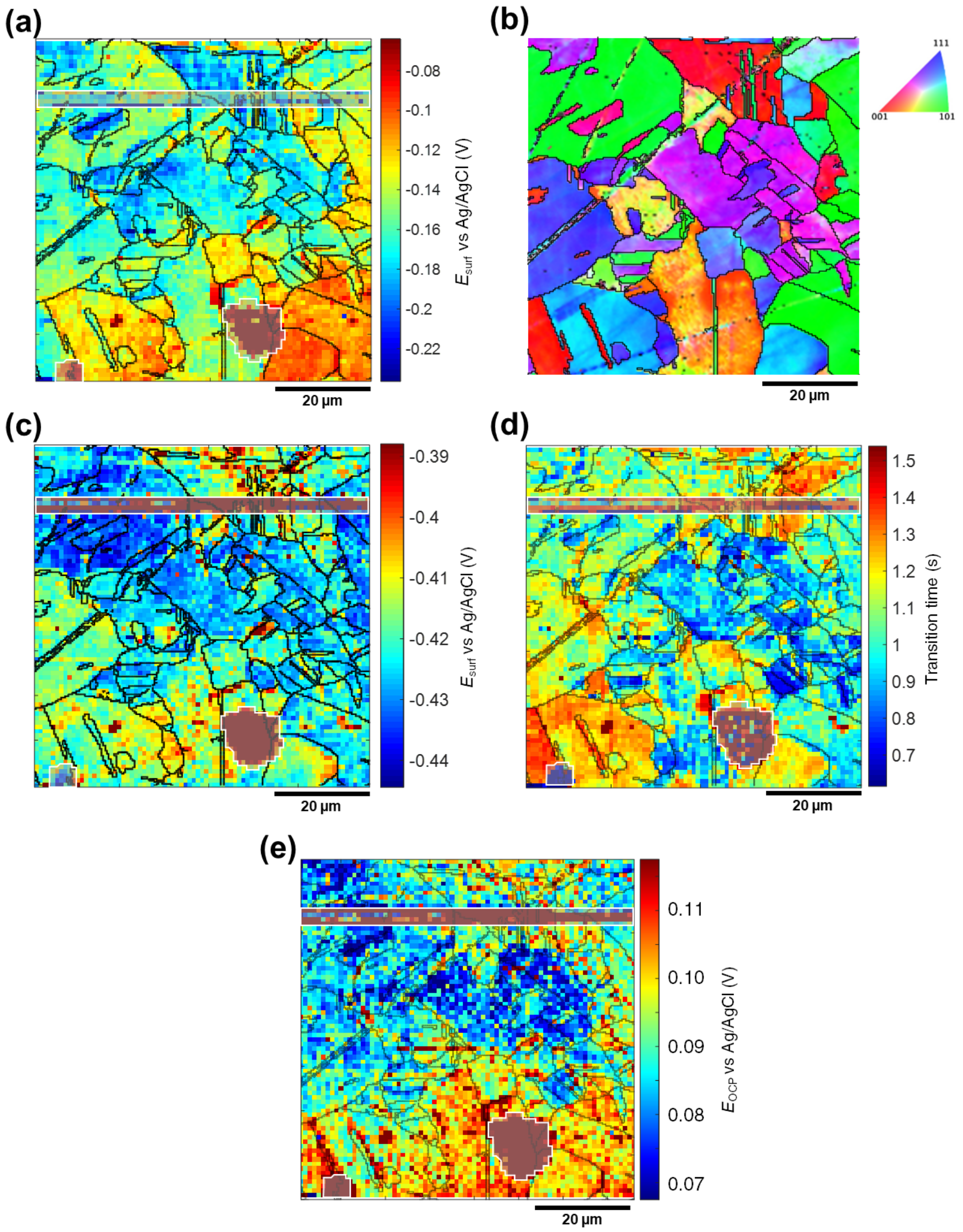

Figure S10. Maps and graphs of grain dependent analysis of the cathodic chronopotentiometric pulse measured in Movie S3. **(a)** E_{surf} map of the Cu reduction (Cu^{2+}/Cu) process on a polycrystalline Cu foil electrode immersed in dodecane, obtained in the SECCM configuration. The map was extracted at time 0.1 s of Movie S3. **(b)** Crystallographic orientation map, IPFz (Inverse Pole Figure normal to the z axis) obtained with EBSD over the same area examined in Movie S3 **(c)** E_{surf} map of the ORR process on a polycrystalline Cu foil electrode immersed in dodecane, obtained in the SECCM configuration. The map was extracted at time 2 s of Movie S3. **(d)** Map of $\tau_{Cu^{2+}/Cu_{(s)}}$, extracted from Movie S3. (e) E_{OCP} map of a polycrystalline Cu foil electrode immersed in dodecane, obtained in the SECCM configuration. The map was extracted at time -0.01 s of Movie S3. During these measurements, the nanopipet probe contained aerated 10 mM H_2SO_4 and contacted an area of 6.4×10^{-9} cm^2, with $I_{app} = -0.88$ mA cm^{-2}. All the grain boundaries from Figure S10b were overlapped to Figure S10a − e. Note that the shaded areas in (a,c-e) were not taken into consideration during the grain analysis, due to image artefacts caused by surface contamination (*e.g.*, dust or dirt), shown above in Figure S3b.

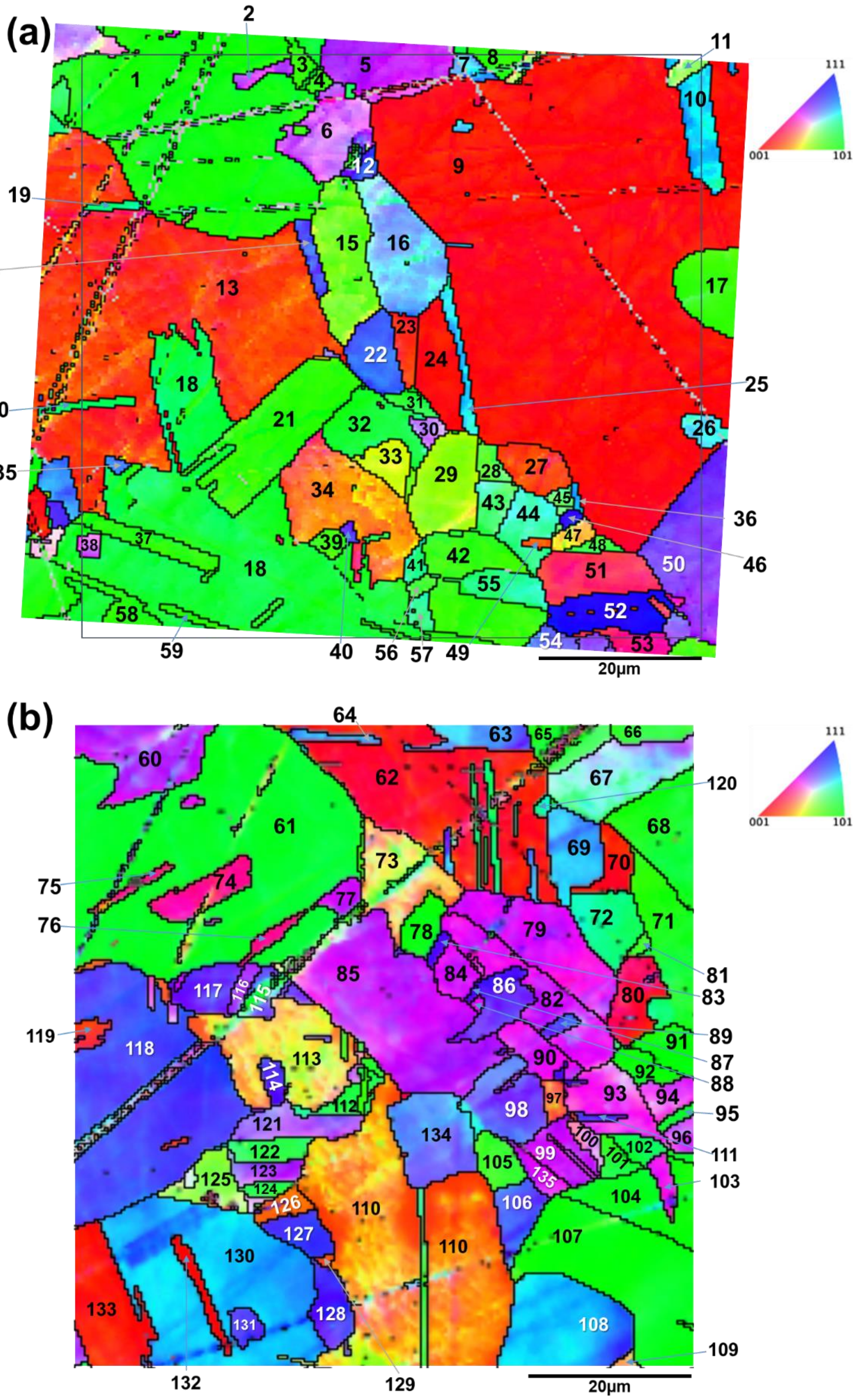

Figure S11. Definition of the grain ID for each grain analysed from **(a)** Movie S2 (*i.e.*, reproduction of the EBSD data shown in the main text, Figure 4b) and **(b)** Movie S3 (*i.e.*, reproduction of the EBSD data shown in the Supporting Information, Figure S10).

Table S3. List of all grains analysed by SECCM (Supporting Information, Movie S2, Movie S3 and Figure S10, and main text, Figure 4 and 5), with the average Euler angles, Miller indices and Projection Coordinates, τ and E_{surf} listed for each one. The grain IDs correspond to those defined in Figure S11.

ID	average Euler angles (˚)			average Miller indices			Projection Coords. (˚)		Average E_{surf} (V vs Ag/AgCl)		Average τ (s)	Average E_{OCP} t = -0.01s (V vs Ag/AgCl)	Size (pix.)
	φ_1	Φ	φ_2	*h*	*k*	*l*	C_1	C_2	Cu depos.	ORR			
1	46.18	42.47	2.72	0.032	0.674	0.738	63.22	2.49	-0.132	-0.437	1.102	0.0097	519
2	305.1	31.93	52.89	0.422	0.319	0.849	43.57	20.87	-0.156	-0.433	0.933	0.0047	15
3	84.07	38.52	88.19	0.622	0.020	0.782	57.89	2.59	-0.128	-0.440	1.087	0.0084	9
4	87.60	37.71	85.96	0.610	0.043	0.791	56.89	3.93	-0.138	-0.440	1.143	0.0085	7
5	279.36	31.39	49.97	0.399	0.335	0.854	42.06	21.56	-0.166	-0.431	1.005	0.0058	61
6	302.65	31.28	56.73	0.434	0.285	0.855	43.92	18.66	-0.163	-0.433	0.933	0.0052	74
7	98.77	40.19	62.97	0.575	0.293	0.764	55.18	21.67	-0.116	-0.402	1.200	0.0153	9
8	202.51	38.94	88.45	0.628	0.017	0.778	58.45	2.37	-0.133	-0.434	1.038	0.0090	6
9	248.33	4.52	57.84	0.067	0.042	0.997	6.89	2.27	-0.164	-0.422	1.203	0.0161	1601
10	332.84	46.80	25.56	0.315	0.658	0.685	60.70	25.23	-0.126	-0.418	1.265	0.0131	40
11	111.00	31.91	75.08	0.511	0.136	0.849	48.36	9.64	-0.143	-0.422	1.260	0.0119	8
12	139.90	42.78	37.70	0.415	0.537	0.734	53.96	31.53	-0.114	-0.419	1.187	0.0124	12
13	41.25	10.79	80.62	0.185	0.031	0.982	16.95	2.93	-0.175	-0.428	1.138	0.0125	668
14	140.50	41.49	35.36	0.383	0.540	0.749	53.69	28.71	-0.129	-0.422	1.190	0.0121	18
15	28.97	32.16	6.26	0.058	0.529	0.847	49.14	5.30	-0.136	-0.437	1.287	0.0111	99
16	122.96	38.51	63.26	0.556	0.280	0.782	53.65	20.26	-0.152	-0.427	1.213	0.0070	118
17	221.31	37.18	1.27	0.013	0.604	0.797	55.95	2.54	-0.124	-0.435	1.098	0.0119	30
18	336.08	43.83	9.51	0.114	0.683	0.721	63.11	8.61	-0.119	-0.430	1.125	0.0154	547
19	245.76	46.01	16.04	0.199	0.691	0.695	62.84	15.75	-0.116	-0.427	1.028	0.0158	6
20	248.06	44.73	12.54	0.153	0.687	0.710	62.96	11.79	-0.133	-0.432	1.056	0.0109	8
21	92.58	38.91	1.31	0.014	0.628	0.778	58.38	2.26	-0.127	-0.438	1.095	0.0120	158
22	143.52	43.20	33.64	0.379	0.570	0.729	55.70	29.14	-0.112	-0.418	1.245	0.0148	58
23	50.62	10.31	71.44	0.170	0.057	0.984	16.15	3.82	-0.165	-0.427	1.125	0.0145	20
24	229.20	3.92	77.25	0.067	0.015	0.998	6.19	1.24	-0.165	-0.424	1.228	0.0158	59
25	332.89	44.17	25.61	0.301	0.628	0.717	58.91	23.40	-0.129	-0.419	1.321	0.0152	27
26	99.51	41.33	65.32	0.600	0.276	0.751	56.88	20.64	-0.123	-0.418	1.239	0.0142	9
27	127.86	11.59	11.30	0.039	0.197	0.980	18.21	3.37	-0.165	-0.427	1.195	0.0153	37
28	44.43	35.70	78.85	0.573	0.113	0.812	53.81	8.24	-0.128	-0.428	1.268	0.0136	18
29	213.55	28.65	2.74	0.023	0.479	0.878	43.82	3.85	-0.125	-0.430	1.352	0.0153	87
30	268.63	32.75	31.08	0.279	0.463	0.841	46.22	18.72	-0.136	-0.425	1.137	0.0121	8
31	49.01	40.33	9.69	0.109	0.638	0.762	59.67	7.91	-0.111	-0.430	1.084	0.0135	13
32	170.60	42.16	81.19	0.663	0.103	0.741	61.79	7.52	-0.117	-0.429	1.143	0.0139	69
33	212.08	25.27	2.79	0.021	0.426	0.904	38.82	3.81	-0.123	-0.417	1.267	0.0159	29
34	36.37	17.95	83.20	0.306	0.036	0.951	27.97	4.04	-0.159	-0.429	1.015	0.0151	150
35	279.63	42.63	58.68	0.579	0.352	0.736	55.99	26.88	-0.099	-0.413	1.224	0.0156	4
36	332.14	46.41	28.79	0.349	0.635	0.689	59.54	27.90	-0.100	-0.407	1.314	0.0198	4
37	55.20	39.44	86.37	0.634	0.040	0.772	59.24	3.50	-0.117	-0.432	1.136	0.0143	33
38	243.44	30.69	56.92	0.428	0.279	0.860	43.28	18.13	-0.122	-0.434	1.059	0.0131	5
39	91.53	36.97	4.27	0.045	0.600	0.799	55.88	4.13	-0.140	-0.437	1.128	0.0117	9

40	138.98	39.82	38.76	0.401	0.499	0.768	51.00	29.09	-0.149	-0.422	1.053	0.0129	3
41	122.32	42.90	72.53	0.649	0.204	0.733	60.22	15.44	-0.103	-0.424	1.060	0.0162	6
42	41.91	37.89	88.80	0.614	0.013	0.789	56.94	2.38	-0.117	-0.432	1.151	0.0170	107
43	314.90	38.27	16.26	0.173	0.595	0.785	56.05	12.44	-0.129	-0.425	1.280	0.0143	25
44	91.12	41.01	69.83	0.616	0.226	0.755	57.85	16.77	-0.122	-0.426	1.212	0.0121	44
45	50.65	36.38	73.64	0.569	0.167	0.805	53.86	11.84	-0.125	-0.425	1.221	0.0142	6
46	201.72	46.12	47.91	0.535	0.483	0.693	54.92	37.91	-0.106	-0.413	1.240	0.0190	6
47	112.91	24.33	77.71	0.403	0.088	0.911	37.67	6.73	-0.153	-0.427	1.136	0.0141	15
48	243.17	37.82	79.46	0.603	0.112	0.790	56.58	8.15	-0.124	-0.428	1.177	0.0158	11
49	295.90	12.65	5.82	0.022	0.218	0.976	19.78	2.90	-0.163	-0.426	1.249	0.0144	4
50	300.56	36.78	52.23	0.473	0.367	0.801	48.47	25.56	-0.136	-0.422	0.856	0.0132	97
51	179.03	17.72	31.26	0.158	0.260	0.953	26.46	9.19	-0.158	-0.427	0.907	0.0143	55
52	106.23	48.94	45.28	0.536	0.531	0.657	55.86	42.70	-0.084	-0.403	1.167	0.0254	67
53	169.63	19.98	41.00	0.224	0.258	0.940	28.04	12.66	-0.141	-0.428	0.821	0.0173	4
54	319.97	39.16	33.03	0.344	0.529	0.775	52.37	25.03	-0.128	-0.412	1.047	0.0228	2
55	319.44	42.33	17.20	0.199	0.643	0.739	59.82	14.92	-0.114	-0.422	1.206	0.0180	36
56	354.16	36.82	9.74	0.101	0.591	0.801	55.42	7.48	-0.112	-0.425	1.187	0.0182	8
57	121.93	41.02	71.90	0.624	0.204	0.754	58.41	15.06	-0.098	-0.426	1.107	0.0198	16
58	54.94	40.47	86.17	0.648	0.043	0.761	60.60	3.51	-0.107	-0.433	1.140	0.0195	26
59	54.70	39.98	87.58	0.642	0.027	0.766	59.95	2.68	-0.103	-0.433	1.133	0.0210	14
60	328.76	32.32	41.25	0.353	0.402	0.845	42.67	22.87	-0.176	-0.425	1.066	0.0023	101
61	244.98	45.12	82.89	0.703	0.088	0.706	64.70	6.82	-0.151	-0.431	1.105	0.0059	474
62	64.84	7.41	66.92	0.119	0.051	0.992	11.56	3.09	-0.185	-0.414	1.146	0.0092	229
63	287.01	41.94	60.82	0.584	0.326	0.744	56.08	24.65	-0.137	-0.403	1.278	0.0117	24
64	287.01	42.88	61.07	0.596	0.329	0.733	56.93	25.21	-0.125	-0.407	1.269	0.0109	9
65	5.45	41.35	6.59	0.076	0.656	0.751	61.38	5.55	-0.124	-0.404	1.208	0.0115	33
66	232.67	38.51	10.45	0.113	0.612	0.782	57.43	8.19	-0.143	-0.421	1.156	0.0074	18
67	129.55	36.52	65.63	0.542	0.246	0.804	52.22	17.34	-0.156	-0.417	1.259	0.0062	89
68	4.79	42.28	6.96	0.082	0.668	0.740	62.32	5.95	-0.141	-0.426	1.086	0.0065	68
69	156.96	43.53	28.02	0.324	0.608	0.725	57.72	24.98	-0.124	-0.413	1.262	0.0112	33
70	14.33	5.19	27.09	0.041	0.081	0.996	8.04	2.36	-0.176	-0.417	1.113	0.0114	15
71	86.40	41.39	83.23	0.657	0.078	0.750	61.39	5.69	-0.131	-0.429	1.061	0.0089	97
72	99.23	44.99	72.89	0.676	0.208	0.707	61.88	16.20	-0.138	-0.417	1.074	0.0086	59
73	56.95	26.01	73.24	0.420	0.126	0.899	39.91	8.76	-0.160	-0.418	1.110	0.0085	69
74	148.18	20.35	46.50	0.252	0.239	0.938	28.02	13.45	-0.200	-0.432	0.929	-0.0007	42
75	155.00	19.00	39.67	0.208	0.251	0.946	27.00	11.69	-0.184	-0.425	0.961	0.0020	9
76	149.67	18.91	44.55	0.227	0.231	0.946	25.98	12.55	-0.190	-0.428	1.091	0.0042	12
77	330.04	30.71	42.91	0.348	0.374	0.860	40.38	21.97	-0.168	-0.429	0.881	0.0028	15
78	24.83	44.90	2.54	0.031	0.705	0.708	65.79	2.39	-0.138	-0.426	1.143	0.0089	31
79	113.74	29.21	46.73	0.355	0.335	0.873	38.62	20.72	-0.173	-0.426	0.922	0.0027	129
80	84.90	12.31	48.12	0.159	0.142	0.977	17.62	7.52	-0.177	-0.422	1.046	0.0085	46
81	333.40	37.13	6.77	0.071	0.599	0.797	56.06	5.60	-0.136	-0.424	1.221	0.0129	7
82	329.65	29.25	43.37	0.336	0.355	0.872	38.63	20.79	-0.185	-0.427	0.926	0.0029	94
83	296.10	42.80	45.03	0.481	0.480	0.734	51.05	35.38	-0.166	-0.419	0.915	0.0053	6
84	117.96	29.63	42.72	0.335	0.363	0.869	39.26	20.92	-0.178	-0.429	0.901	0.0027	26
85	329.90	31.31	41.57	0.345	0.389	0.854	41.47	22.06	-0.173	-0.427	0.966	0.0037	272
86	151.42	39.73	40.73	0.417	0.484	0.769	50.17	30.02	-0.152	-0.419	1.087	0.0063	38

87	295.60	44.61	44.05	0.488	0.505	0.712	52.91	37.11	-0.150	-0.409	1.105	0.0097	2
88	117.81	28.22	45.24	0.336	0.333	0.881	37.05	20.27	-0.165	-0.425	0.919	0.0052	4
89	144.28	40.99	50.38	0.505	0.418	0.755	51.72	30.78	-0.148	-0.416	1.159	0.0081	8
90	113.27	29.22	48.09	0.363	0.326	0.873	39.05	20.30	-0.173	-0.429	0.865	0.0015	32
91	164.62	43.56	0.22	0.003	0.689	0.725	64.64	0.56	-0.126	-0.429	1.074	0.0098	31
92	235.74	44.93	89.01	0.706	0.012	0.708	66.23	0.92	-0.120	-0.426	1.098	0.0121	22
93	173.02	28.03	46.76	0.342	0.322	0.883	37.31	19.66	-0.155	-0.426	0.974	0.0066	61
94	325.13	29.06	37.35	0.295	0.386	0.874	40.20	18.58	-0.145	-0.422	0.940	0.0085	21
95	237.52	45.02	83.96	0.703	0.074	0.707	64.93	5.76	-0.139	-0.418	0.983	0.0104	6
96	325.88	30.54	37.01	0.306	0.406	0.861	42.02	19.66	-0.138	-0.425	0.971	0.0093	13
97	24.86	17.78	18.72	0.098	0.289	0.952	27.60	6.55	-0.157	-0.421	1.098	0.0113	14
98	154.45	38.95	33.92	0.351	0.522	0.778	51.88	25.41	-0.135	-0.412	1.093	0.0088	66
99	325.98	30.90	45.27	0.365	0.361	0.858	39.98	22.73	-0.153	-0.426	0.809	0.0053	36
100	124.80	29.40	28.96	0.238	0.430	0.871	42.69	15.43	-0.126	-0.415	0.930	0.0105	10
101	24.56	40.57	81.91	0.644	0.092	0.760	60.23	6.67	-0.109	-0.421	1.128	0.0125	13
102	257.72	45.24	10.72	0.132	0.698	0.704	63.80	10.30	-0.120	-0.423	0.988	0.0111	16
103	73.73	27.33	47.90	0.341	0.308	0.888	36.84	18.72	-0.137	-0.424	0.764	0.0078	14
104	161.80	44.00	3.17	0.038	0.694	0.719	64.87	2.81	-0.108	-0.421	1.035	0.0127	76
105	69.20	37.74	82.58	0.607	0.079	0.791	56.83	6.02	-0.124	-0.428	1.151	0.0110	29
106	149.70	41.64	35.10	0.382	0.544	0.747	53.91	28.68	-0.116	-0.405	1.205	0.0113	33
107	232.44	42.71	3.34	0.040	0.677	0.735	63.44	2.95	-0.113	-0.421	1.047	0.0125	224
108	9.91	46.92	62.06	0.645	0.342	0.683	60.11	27.52	-0.103	-0.417	1.197	0.0125	136
109	297.51	23.06	73.30	0.375	0.113	0.920	35.61	7.81	-0.118	-0.417	1.010	0.0151	7
110	48.34	19.33	83.03	0.329	0.040	0.944	30.08	4.31	-0.151	-0.415	1.087	0.0133	395
111	355.10	43.32	50.83	0.532	0.433	0.728	53.86	33.02	-0.126	-0.415	0.868	0.0103	7
112	56.81	41.82	12.47	0.144	0.651	0.745	60.58	10.59	-0.132	-0.425	1.084	0.0103	30
113	52.53	25.26	76.23	0.414	0.102	0.904	39.00	7.46	-0.182	-0.430	1.107	0.0075	145
114	138.09	41.93	49.55	0.508	0.434	0.744	52.21	32.24	-0.173	-0.415	1.098	0.0050	14
115	309.15	38.26	29.39	0.304	0.540	0.785	52.64	21.90	-0.174	-0.425	1.167	0.0048	29
116	110.26	33.40	52.23	0.435	0.337	0.835	44.97	22.43	-0.173	-0.425	0.994	0.0034	13
117	299.96	39.30	40.04	0.407	0.485	0.774	50.04	29.22	-0.186	-0.422	1.143	0.0039	36
118	90.36	41.96	53.53	0.538	0.397	0.744	53.71	29.90	-0.158	-0.416	1.156	0.0063	339
119	217.01	11.38	73.11	0.189	0.057	0.980	17.84	4.00	-0.183	-0.417	1.172	0.0075	13
120	102.59	45.73	69.79	0.672	0.247	0.698	61.52	19.53	-0.136	-0.410	1.217	0.0061	4
121	321.42	33.94	56.65	0.466	0.307	0.830	46.93	20.79	-0.162	-0.422	0.976	0.0062	36
122	55.89	40.23	12.13	0.136	0.631	0.763	59.07	9.84	-0.140	-0.425	1.105	0.0100	29
123	321.49	33.60	53.64	0.446	0.328	0.833	45.64	21.97	-0.161	-0.424	0.863	0.0045	19
124	56.20	40.87	11.31	0.128	0.642	0.756	59.92	9.34	-0.141	-0.426	1.021	0.0072	8
125	215.50	31.46	12.20	0.110	0.510	0.853	47.99	8.13	-0.154	-0.422	1.260	0.0087	42
126	202.14	13.38	87.49	0.231	0.010	0.973	20.79	2.57	-0.175	-0.422	1.227	0.0093	18
127	91.28	44.18	51.29	0.544	0.436	0.717	54.72	33.66	-0.129	-0.410	1.123	0.0106	31
128	64.07	42.20	46.93	0.491	0.459	0.741	51.37	33.86	-0.119	-0.407	0.938	0.0121	37
129	207.74	11.43	81.63	0.196	0.029	0.980	17.93	2.97	-0.155	-0.417	1.138	0.0122	4
130	137.67	47.06	27.97	0.343	0.647	0.681	60.19	27.65	-0.123	-0.415	1.233	0.0106	366
131	72.52	41.03	37.90	0.403	0.518	0.754	52.39	29.84	-0.137	-0.413	1.154	0.0108	10
132	51.50	0.58	60.17	0.009	0.005	1.000	0.90	0.28	-0.156	-0.418	1.344	0.0167	17
133	93.38	6.18	19.54	0.036	0.101	0.994	9.71	2.36	-0.166	-0.418	1.334	0.0137	93

134	112.54	29.71	47.46	0.365	0.335	0.869	39.41	20.94	-0.127	-0.419	1.159	0.0102	92
135	325.98	30.90	45.27	0.365	0.361	0.858	39.98	22.73	-0.153	-0.427	0.749	0.0045	18

S.8. Structure-electrochemistry analysis of surface defects: cathodic and anodic processes

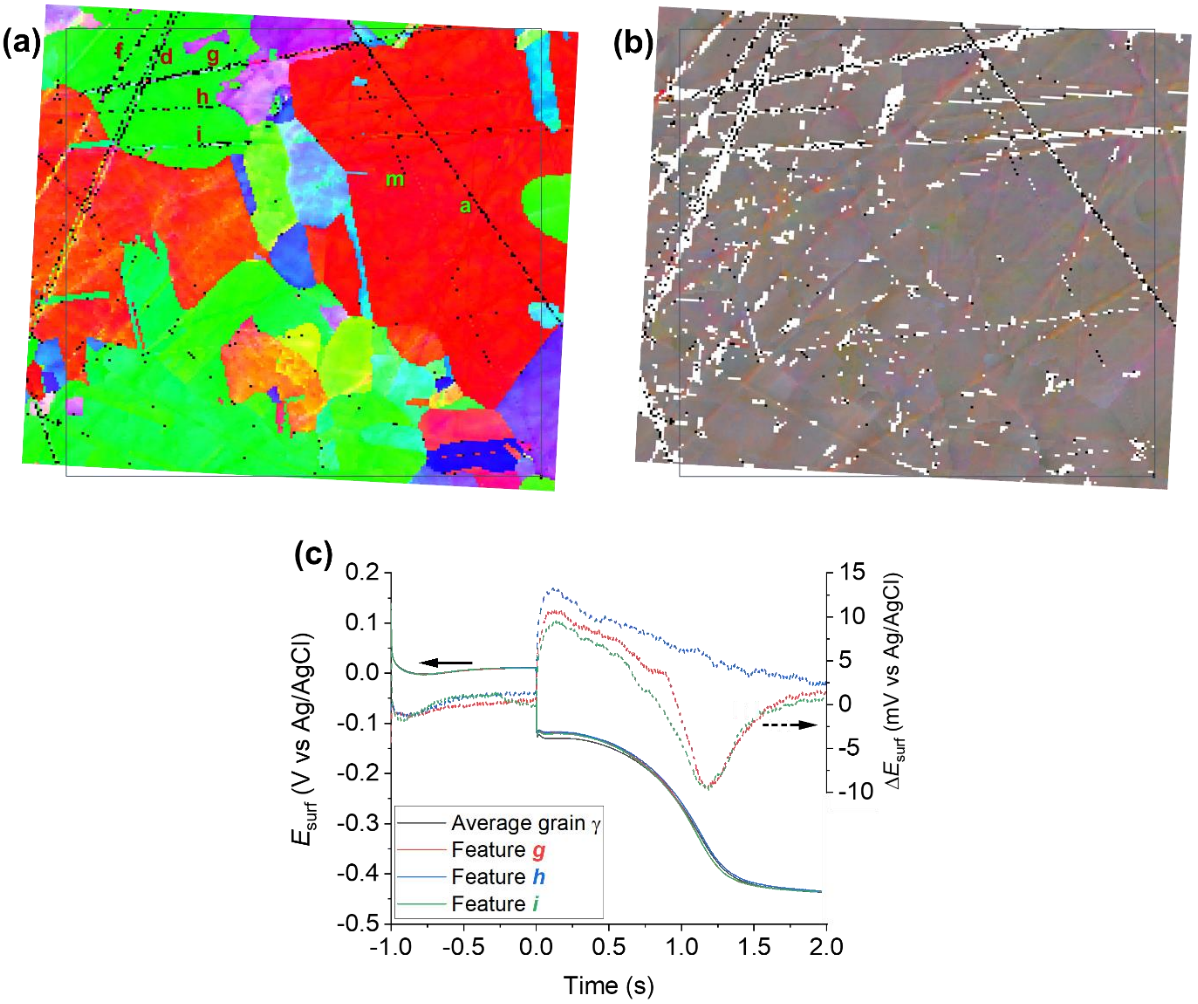

Figure S12. (a) Raw EBSD map (*i.e.*, without grain boundaries drawn) of the area scanned in the ESI, Movie S2. Five main surface defects (scratches), labelled f – i, are identified. Note that scratches can be generally identified as a black zones in EBSD images, due to the fact that the "shadow effect" of the scratches walls generally do not allow the underlying crystallographic orientation to be determined. **(b)** EBSD misorientation colouring map of the same area. The colours in this map indicate inter-pixel misorientation, such that a visible colour difference between any two adjacent points within a given grain represents the misorientation angle between such points. **(c)** Comparison of the average E_{surf}–t curves recorded on the sections of scratches g, h and i (identified in Figure S12a), embedded in grain γ (identified in the main text, Figure 4b), alongside the average curve of grain γ. Each graph plots the average curves (E_{surf}, continuous lines) and, for every scratch, the difference between the features and the grain average potential (ΔE_{surf}, dashed lines).

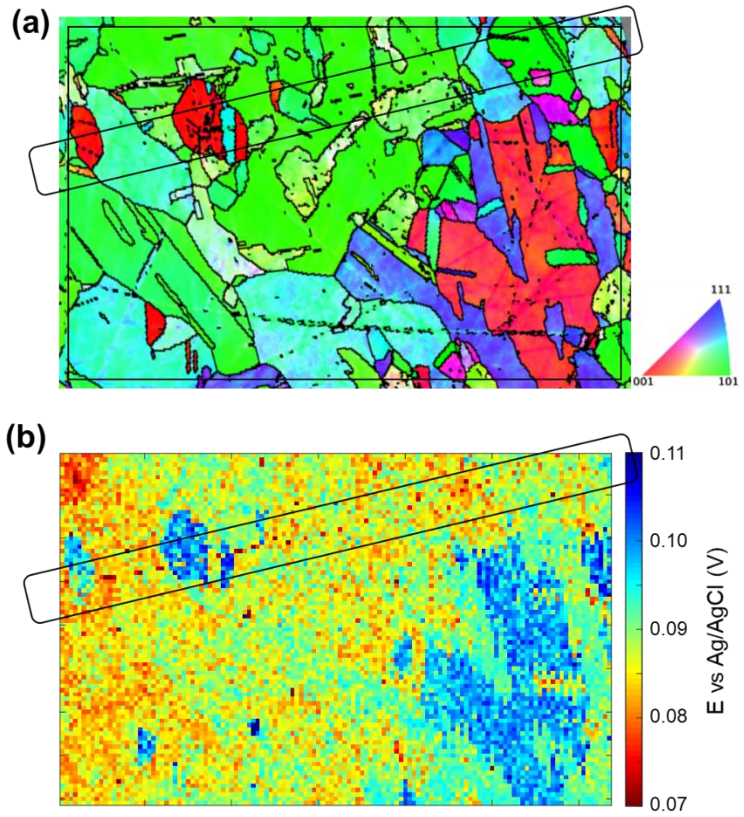

Figure S13. Reproduction of **(a)** the EBSD (IPFz) map and **(b)** the E_{surf} map reported in Figure 3 in the main text (i.e., Cu oxidation activity on polycrystalline Cu). In both images, the marked area contains a visible microscratch, identified in (a), which gives rise to enhanced oxidation activity, visible in (b). Other scratches can also be identified in (a), but they do not appear to give rise to an electrochemical behaviour that is significantly different from underlying grains, in (b).

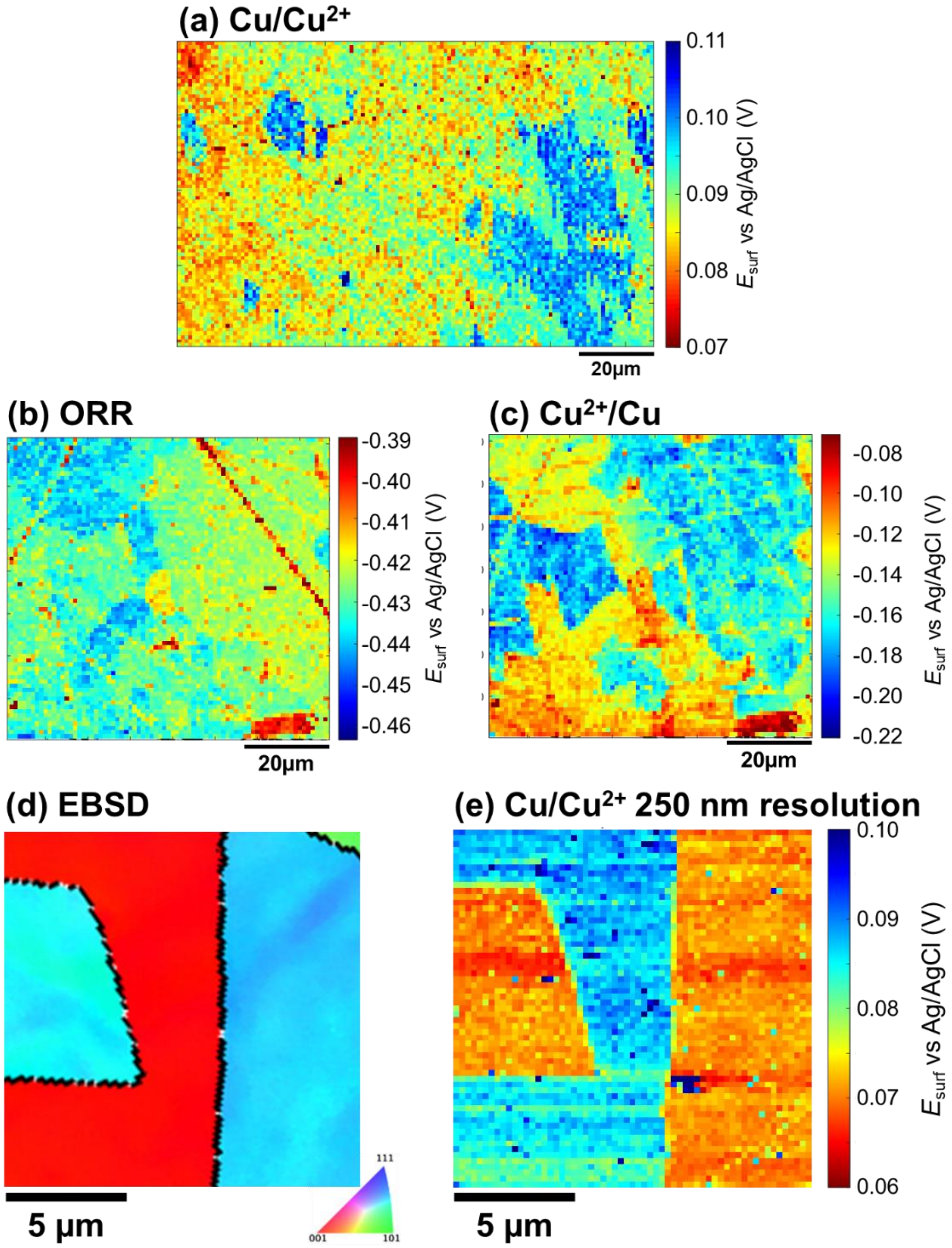

Figure S14. (a-c) Reproduction of, respectively, (a) Figure 3a, (b) Figure 4a and (c) Figure 5a, all without the grain boundaries overlaid on the electrochemical map. (d) EBSD map (IPFz) of a polycrystalline Cu surface and (e) corresponding SECCM E_{surf} map, collected with the following method: a chronopotentiometric pulse of 0.3s at a current of 1pA (≈ 4 mA cm^{-2}, given a contact area of ≈ 180 nm diameter with a probe of ≈ 150 nm diameter), preceded by an OCP step of 0.2s. the map was extracted a time 0.1 s of the anodic pulse.

References

1. J. Ustarroz, I. M. Ornelas, G. Zhang, D. Perry, M. Kang, C. L. Bentley, M. Walker and P. R. Unwin, *ACS Catal.*, 2018, **8**, 6775-6790.
2. W. Xing, M. Yin, Q. Lv, Y. Hu, C. Liu and J. Zhang, in *Rotating Electrode Methods and Oxygen Reduction Electrocatalysts*, eds. W. Xing, G. Yin and J. Zhang, Elsevier, Amsterdam, 2014, pp. 1-31.
3. B. A. Kowert and N. C. Dang, *J. Phys. Chem. A*, 1999, **103**, 779-781.
4. R. Battino and P. G. Seybold, *J. Chem. Eng. Data*, 2011, **56**, 5036-5044.
5. M. H. Abraham and W. E. Acree Jr, *New J. Chem.*, 2004, **28**, 1538-1543.
6. A. L. Barker, J. V. Macpherson, C. J. Slevin and P. R. Unwin, *J. Phys. Chem. B*, 1998, **102**, 1586-1598.
7. D. Kourounis, A. Fuchs and O. Schenk, *IEEE Trans. Power Syst.*, 2018, **33**, 4005-4014.
8. T. N. Andersen, M. H. Ghandehari and H. Eyring, *J. Electrochem. Soc.*, 1975, **122**, 1580-1585.
9. C.-H. Chen, K. E. Meadows, A. Cuharuc, S. C. S. Lai and P. R. Unwin, *Phys. Chem. Chem. Phys.*, 2014, **16**, 18545-18552.
10. R. G. Mariano, K. McKelvey, H. S. White and M. W. Kanan, *Science*, 2017, **358**, 1187.
11. E. Daviddi, Z. Chen, B. Beam Massani, J. Lee, C. L. Bentley, P. R. Unwin and E. L. Ratcliff, *ACS Nano*, 2019, **13**, 13271-13284.
12. M. E. Snowden, A. G. Güell, S. C. S. Lai, K. McKelvey, N. Ebejer, M. A. O'Connell, A. W. Colburn and P. R. Unwin, *Anal. Chem.*, 2012, **84**, 2483-2491.
13. E. Daviddi, K. L. Gonos, A. W. Colburn, C. L. Bentley and P. R. Unwin, *Anal. Chem.*, 2019, **91**, 9229-9237.
